# AdipoR2 recruits protein interactors to promote fatty acid elongation and membrane fluidity

**DOI:** 10.1016/j.jbc.2023.104799

**Published:** 2023-05-08

**Authors:** Mario Ruiz, Ranjan Devkota, Delaney Kaper, Hanna Ruhanen, Kiran Busayavalasa, Uroš Radović, Marcus Henricsson, Reijo Käkelä, Jan Borén, Marc Pilon

**Affiliations:** 1 Department of Chemistry and Molecular Biology, University of Gothenburg, Gothenburg, Sweden; 2 Helsinki University Lipidomics Unit, Helsinki Institute of Life Science, Biocenter Finland, Helsinki, Finland; 3 Molecular and Integrative Biosciences Research Programme, Faculty of Biological and Environmental Sciences, University of Helsinki, Helsinki, Finland; 4 Department of Molecular and Clinical Medicine/Wallenberg Laboratory, Institute of Medicine, University of Gothenburg, Gothenburg, Sweden

**Keywords:** membrane lipid, membrane bilayer, membrane fluidity, fatty acid, phospholipid, acyl-CoA synthetase, adiponectin receptor, PAQR, immunoprecipitation, lipidomics, Laurdan dye, *Caenorhabditis elegans*, HEK293

## Abstract

The human AdipoR2 and its *Caenorhabditis elegans* homolog PAQR-2 are multipass plasma membrane proteins that protect cells against membrane rigidification. However, how AdipoR2 promotes membrane fluidity mechanistically is not clear. Using 13C-labeled fatty acids, we show that AdipoR2 can promote the elongation and incorporation of membrane-fluidizing polyunsaturated fatty acids into phospholipids. To elucidate the molecular basis of these activities, we performed immunoprecipitations of tagged AdipoR2 and PAQR-2 expressed in HEK293 cells or whole *C. elegans*, respectively, and identified coimmunoprecipitated proteins using mass spectrometry. We found that several of the evolutionarily conserved AdipoR2/PAQR-2 interactors are important for fatty acid elongation and incorporation into phospholipids. We experimentally verified some of these interactions, namely, with the dehydratase HACD3 that is essential for the third of four steps in long-chain fatty acid elongation and ACSL4 that is important for activation of unsaturated fatty acids and their channeling into phospholipids. We conclude that AdipoR2 and PAQR-2 can recruit protein interactors to promote the production and incorporation of unsaturated fatty acids into phospholipids.

In animals, most fatty acids in the body, including in cell membranes, are of dietary origin. In human, *de novo* lipogenesis (mostly from liver) accounts for only ∼10% of fatty acids in circulating very-low-density lipoproteins, meaning that ∼90% is diet derived (1, 2). Similarly, in the nematode *Caenorhabditis elegans*, >75% of phospholipid fatty acids are obtained from the diet (3). Critically, local *de novo* lipogenesis and lipid remodeling within each cell can compensate for the varied mixture of fatty acids imported from the circulation. This explains the robustness of membrane composition despite extremely varied diets (4, 5, 6). This homeostasis is necessary since the vital properties of cell membranes, such as packing density, lateral mobility, curvature, and permeability, are greatly influenced by the phospholipid composition (7, 8, 9, 10). Phospholipids with saturated fatty acids (SFAs) pack more densely than those with unsaturated fatty acids (UFAs) of the same length and consequently form more rigid and thicker membranes. Although polyunsaturated fatty acids (PUFAs) are long (18 carbons or more), their multiple double bonds introduce kinks that weaken lateral van der Waals attraction of the acyl chains in phospholipids, thus greatly increasing membrane fluidity. The hydrophilic head groups also impact membrane properties (11). For example, phospholipids with a large choline head group have nearly cylindrical shapes and thus with a small share of the small-head phosphatidylethanolamine can naturally form flat membranes. In contrast, phospholipids with the small ethanolamine head group are conical in shape and therefore form curved membranes.

How do cells robustly maintain their membrane geometry and viscosity given the varied and unpredictable mixture of fatty acids supplied through the circulation? In molecular terms, this robustness depends on protein sensors that monitor membrane properties and can signal to fatty acid synthesis/remodeling enzymes that rectify phospholipid composition and contribute to maintaining the viscosity gradient from the nucleus to the plasma membrane (12, 13). Many of these proteins are evolutionarily conserved and include PCYT1A in the inner nuclear membrane where it promotes phosphatidylcholine synthesis in response to loosening membrane packing (14) and IRE1 in the endoplasmic reticulum (ER) that activates lipogenic pathways in response to thick domains rich in SFAs (15, 16). Human AdipoR2 and its *C. elegans* homolog PAQR-2 are also critical for membrane homeostasis (17, 18, 19, 20, 21, 22, 23, 24, 25, 26). AdipoR2 and PAQR-2 have seven transmembrane domains, with a short extracellular C terminus and a large cytoplasmic N-terminal domain that may regulate access to a hydrolase activity present within a large cytoplasmic-facing cavity (25, 27, 28, 29). Genetic screens in *C. elegans* indicate that a primary function of PAQR-2 is to respond to membrane rigidification by promoting desaturase expression and increasing the PUFA content of phospholipids and that this function is conserved in the human AdipoR2 (17, 18, 19, 22, 23, 26, 30). At least part of the AdipoR2/PAQR-2 downstream cascade relies on a protein-intrinsic ceramidase activity that produces a sphingosine-1 phosphate (S1P) signal that activates the SREBF1/SBP-1 and PPARγ/NHR-49-dependent transcription of desaturases (SBP-1 and NHR-49 are *C. elegans* homologs of SREBF1 and PPARγ, respectively) (31, 32). How AdipoR2/PAQR-2 also promotes PUFA incorporation into phospholipids has not been directly investigated and remains, however, undefined. This is an intriguing question given that fatty acid elongation and desaturation, as well as phospholipid synthesis, take place in the ER while most reports emphasize that AdipoR2/PAQR-2 are present on the plasma membrane, although they are also found in the ER where they could play important roles (29, 33).

Forward genetic screens have important limitations, including the fact that identification of essential genes or genes that are functionally redundant for an important biological process is difficult through screening strategies. Having already performed several forward genetics screens in *C. elegans* to define the PAQR-2 pathway, we felt that other strategies should be deployed. Here, we used a proteomics approach to identify novel PAQR-2 and AdipoR2 interaction partners and discovered that they recruit an evolutionarily conserved fatty acid elongation complex that can channel polyunsaturated fatty acids into phospholipids and in this way contribute to membrane homeostasis.

## Results

### AdipoR2 is required for elongation and desaturation of linoleic acid

**Figure 1:**
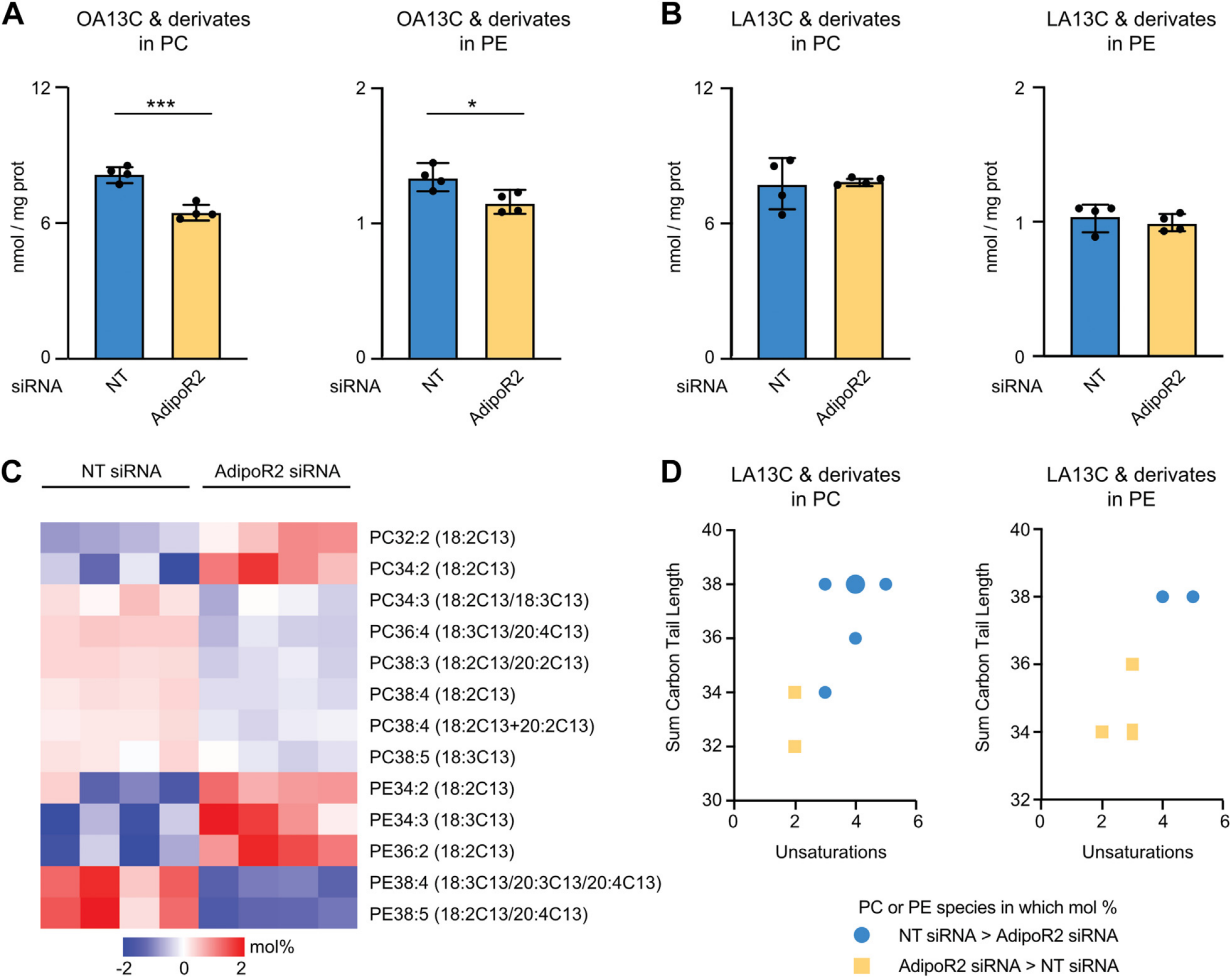
**Fates of 13C-labeled oleic acid (OA13C) and linoleic acid (LA13C) in phospholipids.** HEK293 cells were treated overnight with nontarget (NT) or AdipoR2 siRNA then incubated 6 h in the presence of OA13C or LA13C. The cells were then harvested, and their phospholipid composition was determined by mass spectrometry. *A* and *B*, the levels of incorporated OA13C and its derivatives were reduced in the AdipoR2 siRNA-treated cells; levels of incorporated LA13C were unaffected. *C*, heatmap of 13C-labeled PC and PE species expressed as %mol that were significantly (corrected *p* values *q* < 0.05) different between NT and AdipoR2 siRNA-treated cells. The mean among all samples for each lipid species was adjusted to zero, and the heatmaps report differences from that mean. The 13C-containing fatty acids detected in the phospholipid isobaric species are indicated in parenthesis. Note the depletion of long-chain polyunsaturated species in the AdipoR2 siRNA-treated cells (/, alternatives; +, both acyl chains were labeled). *D*, plots of the degree of unsaturation *versus* total chain length shows that AdipoR2 silencing prevents elongation and further desaturation or incorporation of the exogenously supplied LA13C into PCs and PEs. The larger symbol in *D* indicates that two different PC species had four double bonds and a sum carbon length of 38.

To test whether AdipoR2 influences fatty acid elongation, desaturation, and incorporation of PUFAs into phospholipids, we treated human HEK293 cells with either nontarget siRNA (negative control) or AdipoR2 siRNA, then incubated the cells in the presence of 13C-labeled oleic acid (OA; 18:1n-9) or linoleic acid (LA; 18:2n-6) for 6 h. Samples were then harvested, and their phospholipid composition was determined by mass spectrometry, which allowed detection of 13C-labeled species. In agreement with earlier studies, we found that AdipoR2 siRNA-treated cells have an excess of SFAs in unlabeled phospholipids (see, *e.g.*, PC30:0 and 32:0 in Table S1). We also found that AdipoR2 silencing reduced the levels of 13C-labeled OA and its derivatives in phosphatidylcholines (PCs) and phosphatidylethanolamines (PEs) (Fig. 1*A*) but had no effect on the total levels of 13C-labeled LA in PCs or PEs (Fig. 1*B*). AdipoR2 silencing may therefore reduce the total uptake or incorporation of exogenous OA, but not of LA, into phospholipids. The levels of 13C-labeled LA and its derivates in specific PC and PE species were significantly changed in the AdipoR2 siRNA-treated cells, with a marked excess of short, less desaturated PCs and PEs at the expense of longer more desaturated species derived from exogenously supplied 13C-labeled LA (Fig. 1, *C* and *D*). No such bias was seen when following the fate of 13C-labeled OA: AdipoR2 siRNA-treated cells showed uniformly reduced levels of 13C-labeled species of all lengths and desaturation (Table S1 and Fig. S1). This likely reflects the fact that mammalian cells lack a desaturase capable of introducing a second double bond at the Δ12 position of a fatty acid, and thus neither control nor AdipoR2 siRNA-treated cells can further desaturate OA to LA. Altogether, these results suggest that AdipoR2 contributes to the elongation and further desaturation of LA and/or the incorporation of the resulting long-chain PUFAs into phospholipids.

### Immunoprecipitations identify PAQR-2 and AdipoR2 interactors

**Figure 2:**
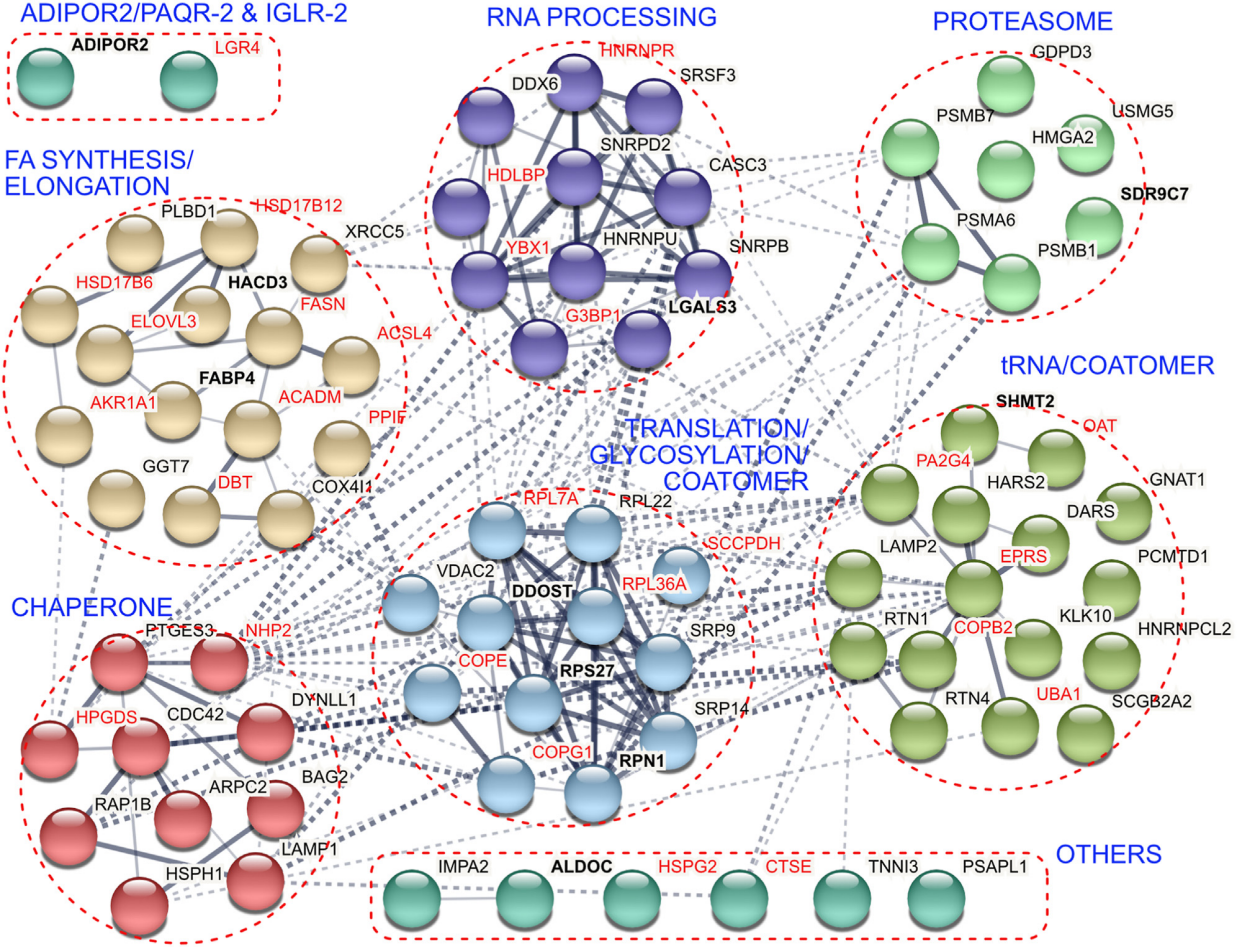
**Summary of HEK293 and worm immunoprecipitation experiments.** Proteins listed here were found in 2/2 HA::PAQR-2 immunoprecipitations (and not in control IPs), or in at least 2/4 HA::AdipoR2 IPs in HEK293 cells (and never in control cells), or were proteins where the homolog was identified at least once in worms and at least once in human. The human proteins and the closest human homolog of worm proteins as per WormBase were used in the STRINGS analysis, with k means clustering set to produce seven clusters differently colored; *BLUE titles* reflect important functions enriched in each cluster. *BOLD text* indicates proteins found both in the human and worm samples, *BLACK text* indicates proteins found only in the human samples, *RED text* indicates proteins detected only in worm samples and for which the name of the human homolog used in the STRING analysis is shown (for example, LGR4 is a human homolog of IGLR-2, which was coimmunoprecipitated with HA::PAQR-2).

We next sought to identify molecular interactors that could explain how AdipoR2/PAQR-2 influences fatty acid elongation and incorporation into phospholipids. An unbiased approach to identify novel protein interactors is purification by immunoprecipitation of protein complexes containing the query protein from mildly lysed cell extracts. We performed immunoprecipitations using an anti-HA antibody from lysates of worms carrying HA::PAQR-2, with a HA tag at the N-terminal end of the endogenous PAQR-2 locus, or of stable clones of HEK293 cells carrying a HA::AdipoR2 expression construct that were generated in a previous study (34). Proteins present in the immunoprecipitated complexes were identified by mass spectrometry. Only proteins that reproducibly and specifically interacted with HA::PAQR-2 or HA::AdipoR2, *i.e.*, proteins that were not present in worms or cells that do not express HA-tagged proteins, were further investigated. These proteins are listed in Table 1 (human proteins) and Table 2 (worm proteins); the complete proteomics data are publicly available at ProteomeXchange (http://www.proteomexchange.org) with identifier PXD031395. The STRING database (35), which mines many sources of information for proteins, was then used to analyze the identified proteins and assign them into clusters (Fig. 2). Three main conclusions can be drawn from the analysis of the AdipoR2/PAQR-2 interactors. First, IGLR-2 was reproducibly detected as a PAQR-2 interaction partner in *C. elegans*, in agreement with our previous genetic interaction studies (19, 25), bifluorescence complementation (BiFC) (19), and fluorescence resonance energy transfer experiments (30). No IGLR-2 mammalian ortholog has yet been identified, and no IGLR-2 homologs were identified in the AdipoR2 immunoprecipitations. Second, several complexes important for the life cycle of transmembrane proteins were found associated with both HA::AdipoR2 and HA::PAQR-2. The fact that the HA tag is present at the N terminus of AdipoR2 and PAQR-2 means that even nascent polypeptides being synthesized can be purified. Indeed, HA::AdipoR2 and HA::PAQR-2 were associated with multiple proteins involved in RNA processing, translation, protein modification (glycosylation), degradation (proteasome components), and coatomer-dependent trafficking. Third, and most interestingly, we found that HA::PAQR-2 and/or HA::AdipoR2 were associated with fatty acid metabolism enzymes including the multifunctional fatty acid synthetase FASN-1 (homologous to human FASN), the elongase ELO-2 (homologous to human ELOVL3/6), the dehydrogenase LET-767 (homologous to human HSD17B12) and fatty acid CoA synthetase ACS-4 (homologous to human ACSL4) in *C. elegans* samples, and the fatty acid binding protein FABP4 (homologous to worm LBP-5/6) and the very-long-chain (3R)-3-hydroxyacyl-CoA dehydratase 3 HACD3 (homologous to *C. elegans* HPO-8) in both the worm and HEK293 samples.

**Table 1:** Human proteomics summary

No	Human	Description of human proteins
1	AdipoR2	Adiponectin receptor protein 2
2	ALDOC	Fructose-bisphosphate aldolase C
3	ARPC2	Actin-related protein 2/3 complex subunit 2
4	BAG2	BAG family molecular chaperone regulator 2
5	CASC3	Protein CASC3
6	CDC42	Cell division control protein 42 homolog
7	COX4I1	Cytochrome c oxidase subunit 4 isoform 1, mitochondrial
8	DARS	Aspartate–tRNA ligase, cytoplasmic
9	DDOST	Dolichyl-diphosphooligosaccharide–protein glycosyltransferase
10	DYNLL1	Dynein light chain 1, cytoplasmic
11	FABP4	Fatty acid–binding protein, adipocyte
12	GDPD3	Glycerophosphodiester phosphodiesterase domain-containing protein 3
13	GGT7	Gamma-glutamyltransferase 7
14	GNAT1	Guanine nucleotide-binding protein G(t) subunit alpha-1
15	HACD3	Very-long-chain (3R)-3-hydroxyacyl-CoA dehydratase 3 (aka PTPLAD1)
16	HARS2	Probable histidine–tRNA ligase, mitochondrial
17	HMGA2	High mobility group protein HMGI-C
18	HNRNPCL2	Heterogeneous nuclear ribonucleoprotein C-like 2
19	HNRNPU	Heterogeneous nuclear ribonucleoprotein U
20	HSPH1	Heat shock protein 105 kDa
21	IMPA2	Inositol monophosphatase 2
22	KLK10	Kallikrein-10
23	LAMP1	Lysosome-associated membrane glycoprotein 1
24	LAMP2	Lysosome-associated membrane glycoprotein 2
25	LGALS3	Galectin-3 OS=*Homo sapiens*
26	PCMTD1	Protein-L-isoaspartate O-methyltransferase domain-containing protein 1
27	PLBD1	Phospholipase B-like 1
28	PSAPL1	Proactivator polypeptide-like 1
29	PSMA6	Proteasome subunit alpha type 6
30	PSMB1	Proteasome subunit beta type 1
31	PSMB7	Proteasome subunit beta type 7
32	PTGES3	Prostaglandin E synthase 3
33	RAP1B	Ras-related protein Rap-1b
34	RPL22	60S ribosomal protein L22
35	RPN1	Dolichyl-diphosphooligosaccharide–protein glycosyltransferase subunit 1
36	RPS27	40S ribosomal protein S27
37	RTN1	Reticulon-1
38	RTN4	Reticulon-4
39	SCGB2A2	Mammaglobin-A
40	SDR9C7	Short-chain dehydrogenase/reductase family 9C member 7
41	SHMT2	Serine hydroxymethyltransferase, mitochondrial
42	SNRPB	Small nuclear ribonucleoprotein-associated proteins B and B′
43	SNRPD2	Small nuclear ribonucleoprotein Sm D2
44	SRP14	Signal recognition particle 14-kDa protein
45	SRP9	Signal recognition particle 9-kDa protein
46	SRSF3	Serine/arginine-rich splicing factor 3
47	TNNI3	Troponin I, cardiac muscle
48	USMG5	Upregulated during skeletal muscle growth protein 5
49	VDAC2	Voltage-dependent anion-selective channel protein 2
50	XRCC5	X-ray repair cross-complementing protein 5

The human proteins listed here were found with a false discovery rate <1% in at least 2/4 HA::AdipoR2 IPs in HEK293 cells (and never in control cells), or were proteins where the homolog was identified at least once in worms and at least once in human.

**Table 2:** *C. elegans* proteomics summary

No	Worm (human)	Description of worm proteins
1	ACDH-8 (ACADM)	Acyl CoA Dehydrogenase
2	ACS-4 (ACSL4)	Fatty acid CoA synthetase family
3	ALDO-1 (ALDOC)	Fructose-bisphosphate aldolase 1
4	ASP-2 (CTSE)	Aspartyl protease
5	B0491.5 (−)	Uncharacterized protein
6	C14B9.10 (−)	Uncharacterized protein
7	CEY-3 (YBX1)	*C. elegans* Y-box
8	CGH-1 (DDX6)	ATP-dependent RNA helicase cgh-1
9	COPB-2 (COPB2)	Probable coatomer subunit beta'
10	COPE-1 (COPE)	Coatomer subunit epsilon
11	COPG-1 (COPG1)	Probable coatomer subunit gamma
12	CYN-3 (PPIF)	Peptidyl-prolyl *cis*-*trans* isomerase 3
13	DBT-1 (DBT)	Lipoamide acyltransferase
14	DHS-3 (SDR9C7)	Dehydrogenases, short chain
15	EARS-1 (EPRS)	Glutamyl(E) amino-acyl tRNA synthetase
16	ELO-2 (ELOVL3/6)	Elongation of very-long-chain fatty acids protein
17	EXC-15 (AKR1A1)	alditol:NADP+ 1-oxidoreductase activity
18	F20G2.2 (HSD17B6)	Similar to hydroxysteroid 17-beta dehydrogenase 6
19	F42G8.10 (−)	Uncharacterized protein
20	FASN-1 (FASN)	Fatty acid synthase
21	GST-7 (HPGDS)	Probable glutathione S-transferase 7
22	GTBP-1 (G3BP1)	Ras-GTPase-activating protein SH3 (three) domain-binding protein
23	HPO-8 (HACD3)	Very-long-chain (3R)-3-hydroxyacyl-CoA dehydratase hpo-8
24	HRPR-1 (HNRNPR)	HnRNP A1 homolog
25	IGLR-2 (LGR4)	Immunoglobulin domain and leucine-rich repeat–containing protein 2
26	LBP-5 (FABP4)	Fatty acid–binding protein homolog 5
27	LBP-6 (FABP4)	Fatty acid–binding protein homolog 6
28	LEC-1 (LGALS3)	32-kDa beta-galactoside-binding lectin
29	LET-767 (HSD17B12)	Very-long-chain 3-oxooacyl-coA reductase let-767
30	LPD-1 (SCCPDH)	Lipid droplet localized protein
31	MEL-32 (SHMT2)	Serine hydroxymethyltransferase
32	OATR-1 (OAT)	Probable ornithine aminotransferase, mitochondrial
33	OSTB-1 (DDOST)	Dolichyl-diphosphooligosaccharide–protein glycosyltransferase 48-kDa subunit
34	PAQR-2 (AdipoR2)	Progestin and AdipoQ Receptor family
35	PUD-1.2 (−)	Protein upregulated in Daf-2(Gf)
36	PUD-3 (−)	Protein upregulated in Daf-2(Gf)
37	RIBO-1 (RPN1)	Dolichyl-diphosphooligosaccharide–protein glycosyltransferase subunit 1
38	RPL-36.A (RPL36A)	Ribosomal protein rpl-41
39	RPL-43 (RPL7A)	60S ribosomal protein L37a
40	RPS-27 (RPS27)	40S ribosomal protein S27
41	UBA-1 (UBA1)	UBA (human ubiquitin) related
42	UNC-52 (HSPG2)	Extracellular matrix protein related to heparan sulfate proteoglycan 2
43	VGLN-1 (HDLBP)	ViGiLN homolog
44	W08E12.7 (PA2G4)	Protein with nucleic acid binding activity similar to proliferation associated 2G4
45	Y48A6B.3 (NHP2)	Putative H/ACA ribonucleoprotein complex subunit 2-like protein

The worm proteins listed here were found with a false discovery rate <1% in 2/2 HA::PAQR-2 immunoprecipitations or were proteins where the homolog was identified at least once in worms and at least once in human. The closest human homolog as per WormBase is listed in parenthesis.

### AdipoR2/PAQR-2 interacts with HACD3/HPO-8

*De novo* synthesis of long-chain fatty acids in animals begins with the cytoplasmic enzyme FASN that produces palmitate (16:0) from malonyl-CoA and acetyl-CoA *via* seven cycles through its multiple enzymatic sites (36). Palmitate is then delivered to an ER complex of four enzymes responsible for the four steps of fatty acid elongation: the rate-limiting fatty acid elongase (one of ELOVL1-7 in human), a 3-ketoacyl-CoA reductase (*e.g.*, HSD17B12 in human), a 3-hydroxyacyl-CoA dehydratase (one of HACD1-4 in human), and a 2,3-*trans*-enoyl-CoA reductase (TECR in human) (37, 38, 39, 40, 41). Strikingly, three of the four enzymes coimmunoprecipitated with AdipoR2 and/or PAQR-2, namely, ELOVL3/6 (ELO-2 in worms), HSD17B12 (LET-767 in worms), and HACD3 (HPO-8 in worms) (Tables 1 and 2; Fig. 2). This strongly suggests that recruitment of this complex contributes to AdipoR2/PAQR-2-mediated membrane homeostasis.

**Figure 3:**
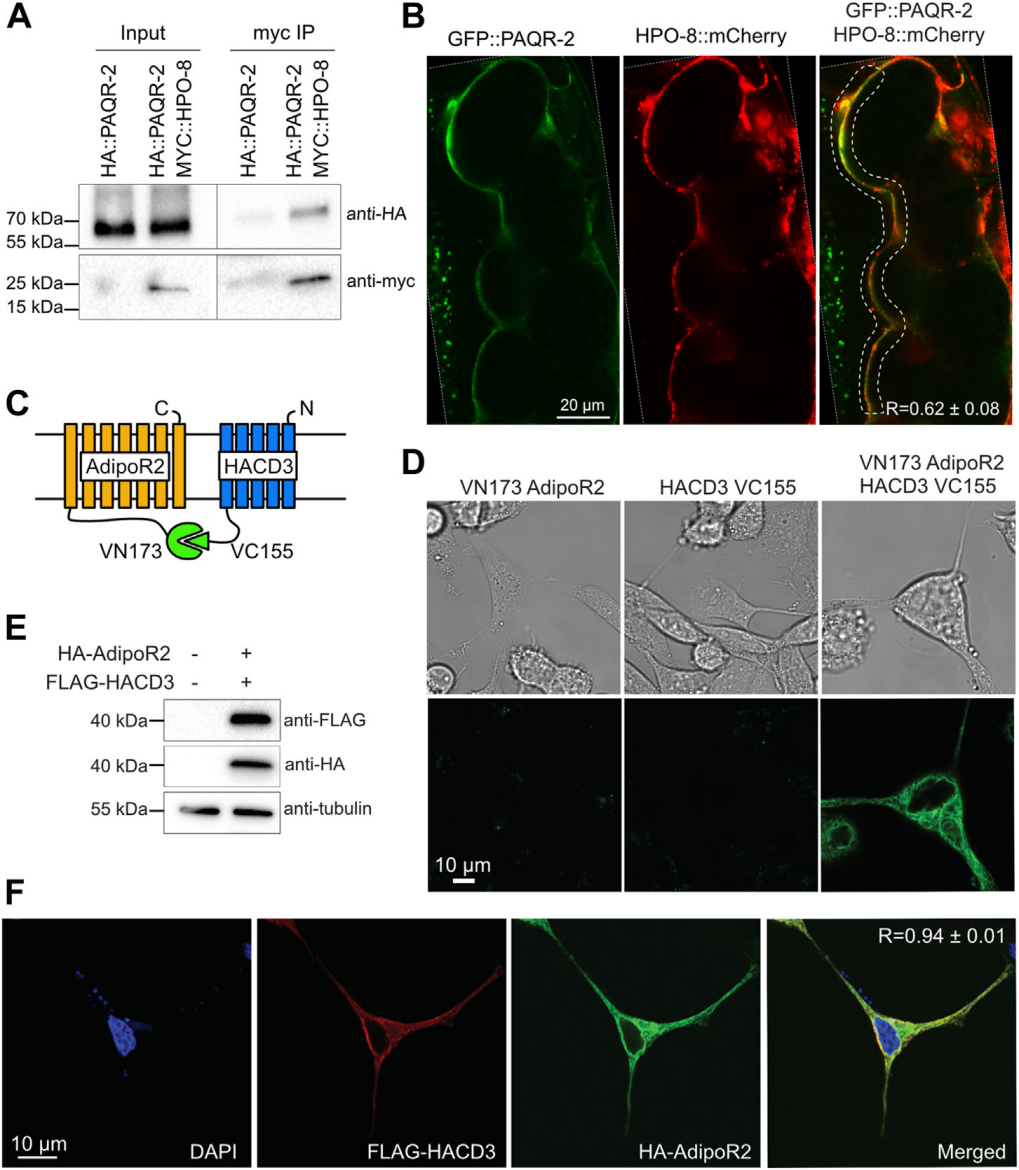
**Confirmation of the interaction between AdipoR2/PAQR-2 and HACD3/HPO-8.***A*, coimmunoprecipitation of HA::PAQR-2 and MYC::HPO-8. *B*, colocalization of GFP::PAQR-2 and HPO-8::mCherry. R is the Pearson coefficient of coexpression within the boxed region (the actual images are contained within the dashed white lines). *C*, cartoon summarizing the BiFC experiment. *D*, BiFC experiment confirming that AdipoR2 interacts with HACD3. *E*, Western blot confirming the transient expression of HA-AdipoR2 and FLAG-HACD3. *F*, immunofluorescence confirming that HA-AdipoR2 colocalizes with FLAG-HACD3. R is the Pearson correlation coefficient between the two fluorophores ± standard deviation (n = 5).

Here, we further characterized HACD3 and its worm homolog HPO-8 because they were found in immunoprecipitations of both HA::AdipoR2 and of HA::PAQR-2, respectively (Tables 1 and 2). We verified the PAQR-2/HPO-8 interaction using two methods: first, reverse immunoprecipitation from worms genetically modified to express a MYC::HPO-8 protein confirmed the association with HA::PAQR-2 (Fig. 3*A*), and second, we observed significant colocalization of GFP::PAQR-2 and HPO-8::mCherry translational reporters in double transgenic worms (Fig. 3*B*). The expression pattern of HPO-8::mCherry appeared normal in the *paqr-2* mutant, suggesting that its expression and localization is mostly independent on PAQR-2 (Fig. S3). We then used BiFC complementation in HEK293 cells (Fig. 3*C*) and found that AdipoR2 interacts with HACD3, producing a signal at the plasma membrane as well as in internal membranes that are likely ER (Figs. 3*D* and S4*A*). In addition, transient coexpression of tagged proteins (Fig. 3*E*) followed by immunofluorescence confirms significant AdipoR2 colocalization with HACD3 (Figs. 3*F* and S4*B*). AdipoR1 and AdipoR2 are known to colocalize (33), which we confirmed and used as a positive control in these experiments (Fig. S2*A*). Altogether, these results suggest that PAQR-2 and AdipoR2 can recruit an ER-associated fatty acid elongation complex containing HPO-8 in worms and HACD3 in human cells, respectively.

### AdipoR2/PAQR-2 interacts with ACSL4/ACS-4

ACSL4 is widely expressed, contributes to the biosynthesis of many PUFA-derived fatty acyl-CoAs, and is involved in maintaining the levels of several PUFA-containing phospholipids. ACSL4 preferentially acts on PUFAs (42, 43, 44), such as arachidonic acid and eicosapentaenoic acid, and removal or reduction of ACSL4 activity markedly decreases the levels of PUFA-containing phospholipids (45, 46, 47). The interaction between PAQR-2 and ACS-4 is also particularly interesting since the human homolog is critical for the activation and channeling of UFAs toward incorporation into glycerophospholipids (48), which could be critical to the function of AdipoR2/PAQR-2 in maintaining membrane fluidity. In addition, a forward genetics screen previously showed that mutations in ACS-13, a paralog of ACS-4, can act as suppressors of *paqr-2* mutant phenotypes (23), thus providing independent support for functional interactions between the PAQR-2 protein and fatty acid CoA synthases.

**Figure 4:**
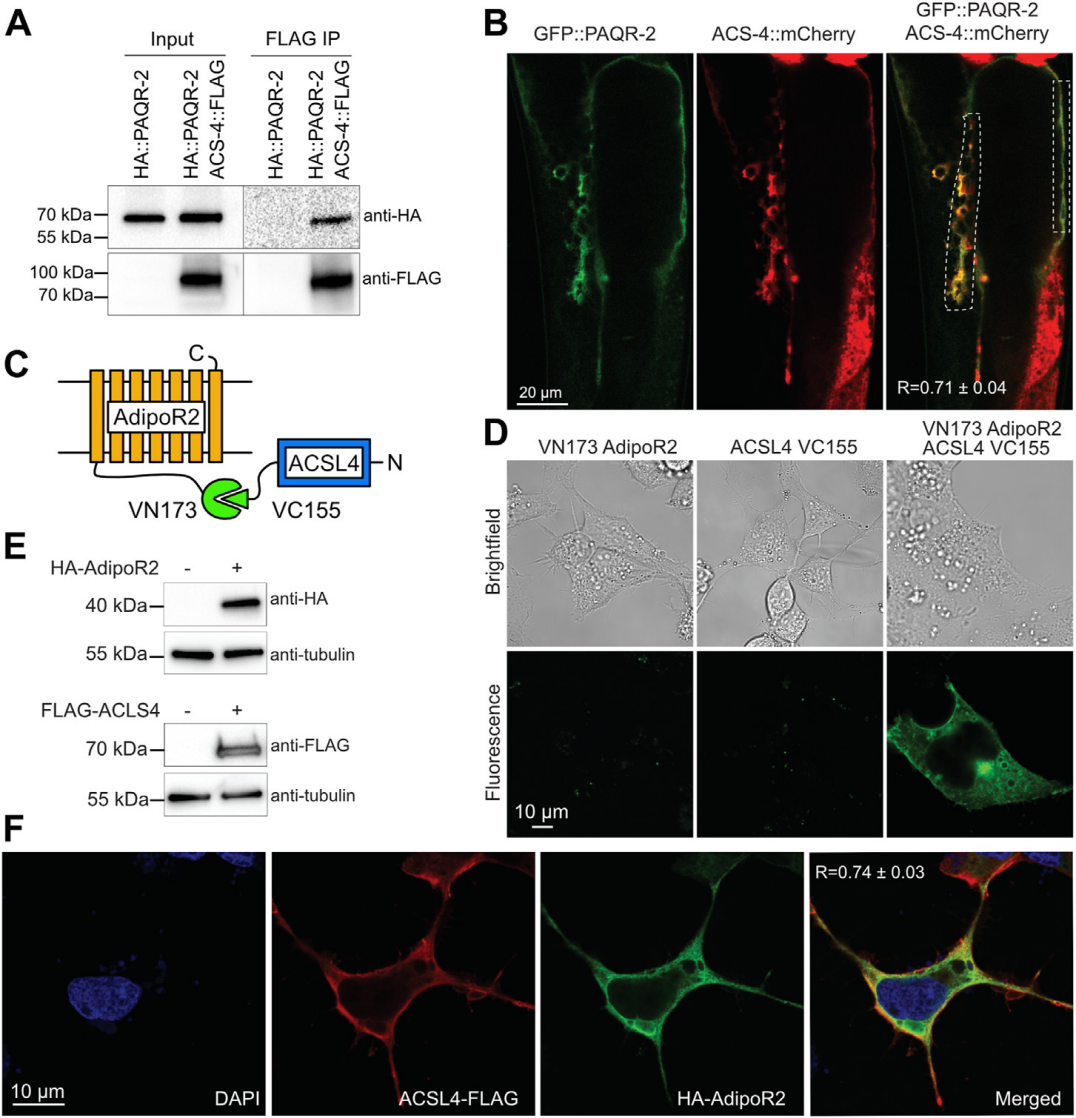
**Confirmation of the interaction between AdipoR2/PAQR-2 and ACSL4/ACS-4.***A*, coimmunoprecipitation of HA::PAQR-2 and ACS-4::FLAG. *B*, colocalization of GFP::PAQR-2 and ACS-4::mCherry. R is the Pearson coefficient of coexpression within the boxed regions. *C*, cartoon summarizing the BiFC experiment. *D*, BiFC experiment confirming that AdipoR2 interacts with ACSL4. *E*, Western blot confirming the transient expression of HA-AdipoR2 and ACSL4-FLAG. *F*, immunofluorescence confirming that HA-AdipoR2 colocalizes with ACSL4-FLAG. R is the Pearson correlation coefficient between the two fluorophores ± standard deviation (n = 5).

Here, we verified the PAQR-2/ACS-4 interaction using two methods: first, reverse immunoprecipitation from worms genetically modified to express a ACS-4::FLAG protein confirmed the association with HA::PAQR-2 (Fig. 4*A*), and second, we observed significant colocalization of GFP::PAQR-2 and ACS-4::mCherry translational reporters in double transgenic worms (Fig. 4*B*). The expression pattern of an ACS-4::GFP translational reporter appeared normal in the *paqr-2* mutant, suggesting that its expression and localization also is mostly independent of PAQR-2 (Fig. S5). We then used bifluorescence complementation in HEK293 cells (Fig. 4*C*) and found that AdipoR2 also interacts with ACSL4, producing a signal at the plasma membrane as well as in internal membranes (Figs. 4*D* and S6*A*). In addition, transient coexpression of tagged proteins (Fig. 4*E*) followed by immunofluorescence confirms significant AdipoR2 colocalization with ACSL4 (Figs. 4*F* and S6*B*). Altogether, these results suggest that PAQR-2 and AdipoR2 can recruit ACS-4 and ACSL4 in worms and HACD3 in human cells, respectively, to activate and channel long-chain PUFAs for incorporation into phospholipids.

### PAQR-2, HPO-8, and ACS-4 are part of one complex

**Figure 5:**
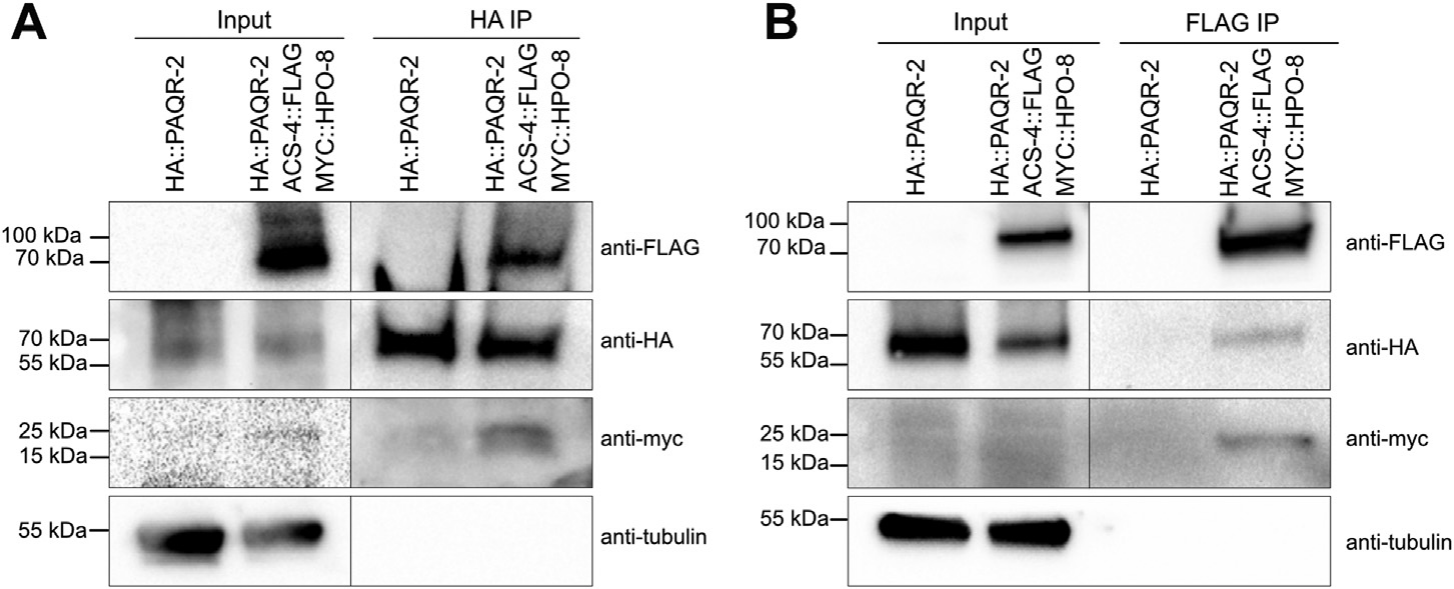
**Coimmunoprecipitations indicate that PAQR-2, HPO-8, and ACS-4 form one complex.***A*, immunoprecipitation of HA::PAQR-2 copurified MYC::HPO-8 and ACS-4::FLAG but not tubulin. *B*, immunoprecipitation of ACS-4::FLAG copurified HA::PAQR-2 and MYC::HPO-8 but not tubulin.

To address the questions of whether all three proteins are part of one complex *in vivo*, we crossed together worms carrying the CRISPR/Cas9-modified endogenous loci to produce a strain where all three tagged proteins are expressed in the same animals. Immunoprecipitation of HA::PAQR-2 copurified MYC::HPO-8 and ACS-4::FLAG (Fig. 5*A*). Similarly, immunoprecipitation of ACS-4::FLAG copurified HA::PAQR-2 and MYC::HPO-8 (Fig. 5*B*). These results suggest that PAQR-2, HPO-8, and ACS-4 can simultaneously be part of the same complex.

### *hpo-8* but *not acs-4 C. elegans* mutants display strong membrane phenotypes

**Figure 6:**
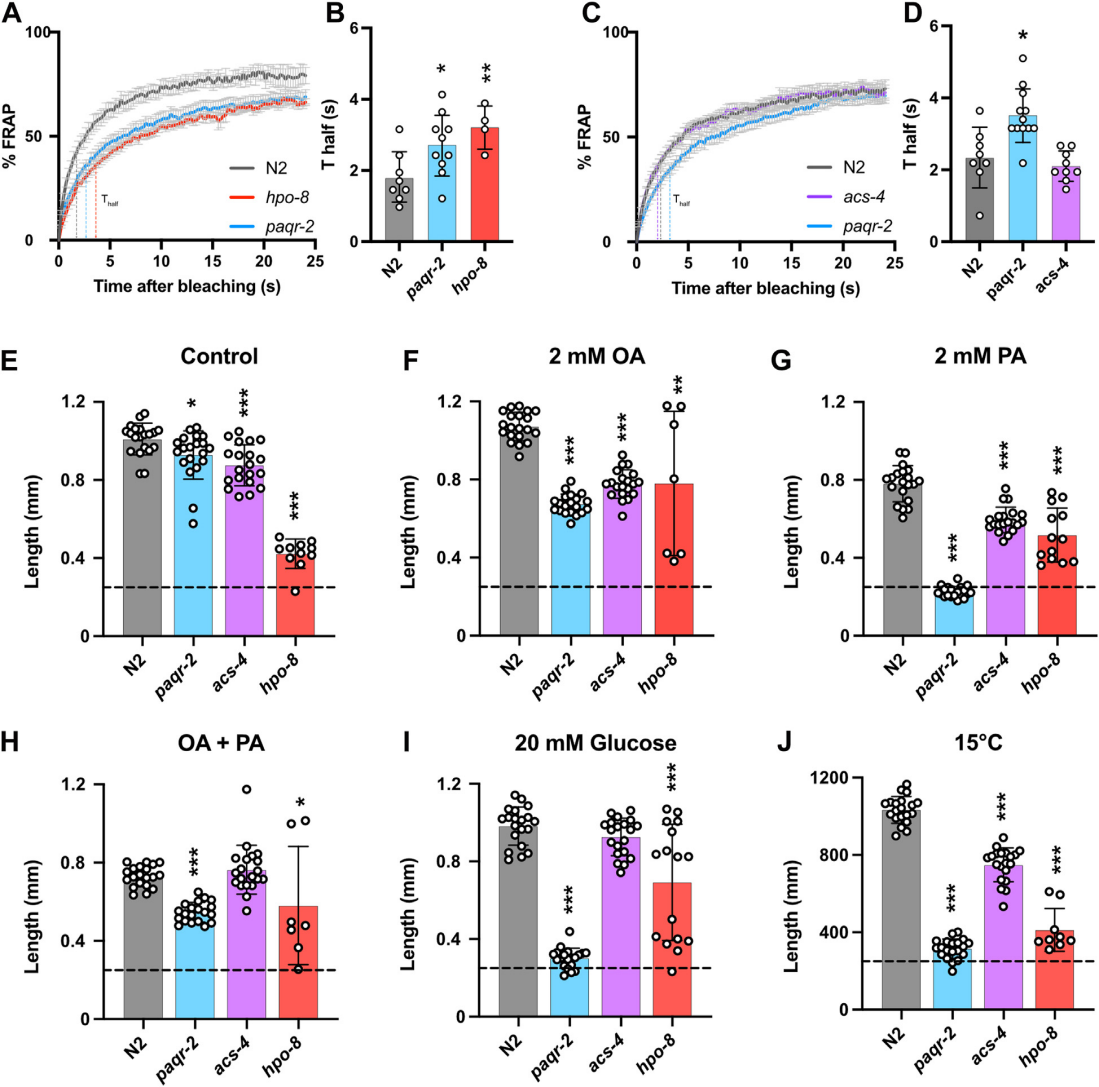
**Membrane and growth properties in the *hpo-8* and *acs-4* mutants.***A*–*D*, FRAP curves and T_half_ values for the *hpo-8* and *acs-4* mutants, respectively, and compared with wildtype N2 and *paqr-2* mutant worms. *E*–*J*, the lengths of worms grown from the L1 stage for 72 h (144 h for the 15 °C experiment) in the indicated condition; the dashed lines indicate the approximate lengths of the synchronized L1s at the start of the growth experiments. Error bars show the standard deviations. ∗*p* < 0.05; ∗∗*p* < 0.01; ∗∗∗*p* < 0.001.

The *hpo-8* mutant is nonviable and the *acs-4* mutant grows well but is sterile (49); these are therefore essential genes, and the mutants are maintained using balancer chromosomes. The immunoprecipitation and colocalization experiments suggest that ACS-4 and HPO-8 may be proteins acting downstream or together with PAQR-2 to maintain membrane homeostasis. To test this hypothesis, we used the method of fluorescence recovery after photobleaching (FRAP) to monitor the lateral mobility of a membrane-bound GFP reporter in living *C. elegans* and found that it is reduced in the intestinal cell membranes of *hpo-8* mutants compared with wildtype worms (Fig. 6, *A* and *B*) and that the *hpo-8* mutant grows poorly in normal medium (Fig. 6*E*) but grows much better in medium supplemented with long-chain fatty acids such OA, palmitic acid (PA), or a combination of both (Fig. 6, *F*–*H*). Inclusion of glucose, which increases the 16:0/18:1 fatty acid ratio in the dietary OP50 *Escherichia coli* (20), also improved the growth of the *hpo-8* mutant (Fig. 6*I*); the *hpo-8* mutant, being unable to produce long fatty acids, likely benefits from any diet in which they are enriched. In contrast, cultivation at 15 °C, considered a membrane-rigidifying low temperature (17), prevented the growth of both *paqr-2* and *hpo-8* mutants. The *acs-4* mutant did not have a significant membrane rigidity phenotype (Fig. 6, *C* and *D*) and grew slightly less well than wildtype worms in all culture conditions tested, except when provided a combination of both OA and PA or when cultivated on glucose plates (Fig. 6, *E*–*J*). There are more than 20 fatty acid CoA synthetases in *C. elegans* (23), and the weaker phenotype of the *acs-4* mutant may reflect functional redundancy.

### HACD3 and ACSL4 maintain membrane fluidity in human cells

**Figure 7:**
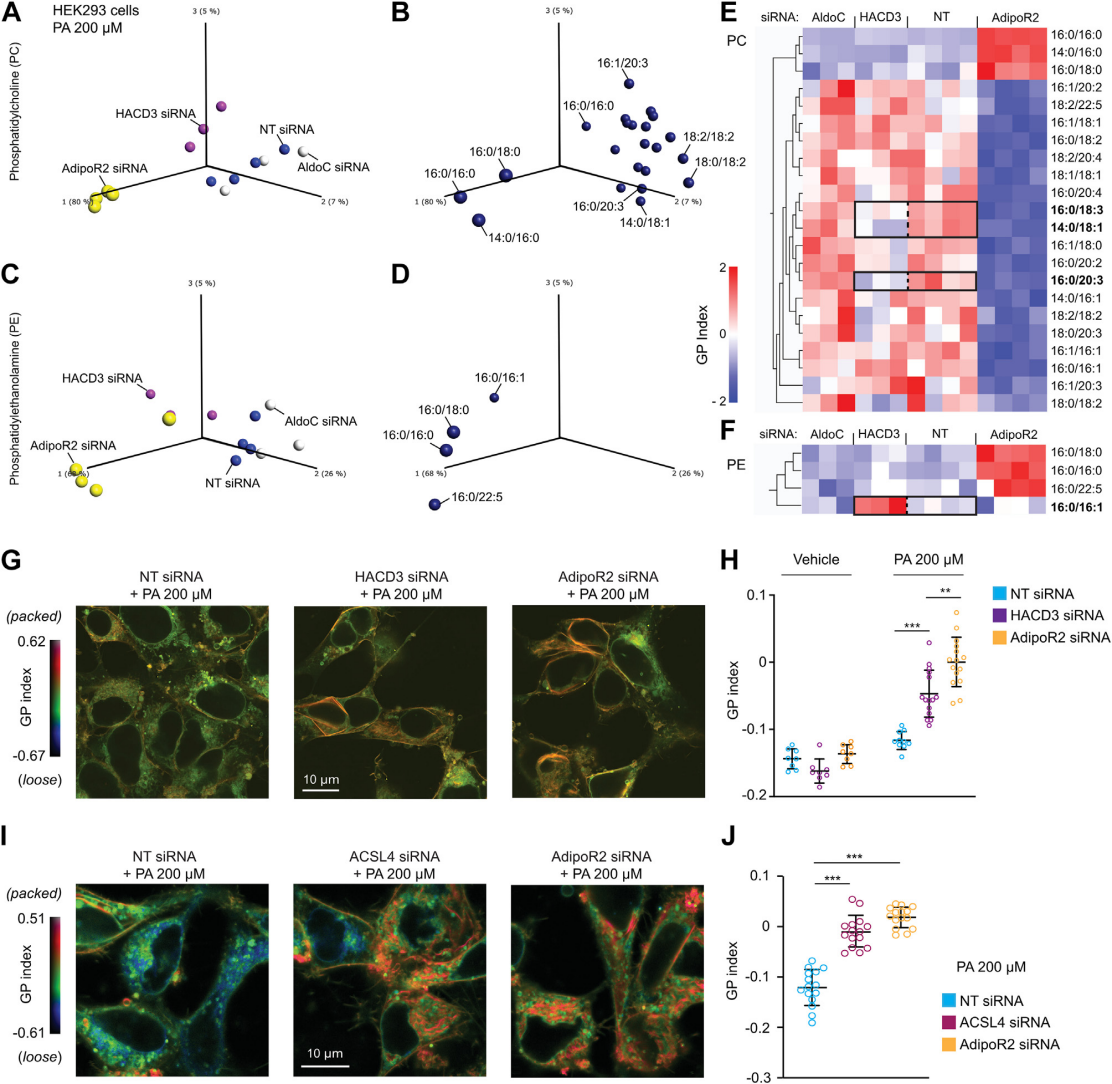
**Lipidomics analysis in HACD3-depleted cells, and membrane fluidity assay in HACD3- and ACSL4-depleted cells.** Principal component analysis scores of the samples (*A*) and underlying phosphatidylcholine (PC) species (*B*) in cells treated with the indicated siRNAs (NT is a nontarget siRNA) and challenged with 200 μM PA; *C* and *D*, similar analyses based on levels of phosphatidylethanolamine (PE) species. *E*, heatmap of the PC species ordered along principal component 1, and highlighting differences between HACD3-depleted and NT cells; *F*, similar heat map for the PE species. *G*–*J*, pseudocolor images and quantification of the global polarization (GP) index of cell treated with the indicated siRNA, challenged with 200 μM PA and stained using Laurdan dye. For *A*–*F*, only lipid species that differed significantly among the groups based on an ANOVA with q < 0.05 were included. Error bars show the standard deviations. ∗∗*p* < 0.01; ∗∗∗*p* < 0.001.

We previously showed that silencing ACSL4 in HEK293 cells results in a slight but significant excess of SFAs in both PCs and PEs (50), which is consistent with the role of ACSL4 in channeling UFAs into phospholipids (48, 50). Here, we show that silencing HACD3 using siRNA in PA-challenged HEK293 cells also alters the composition of phospholipids, as evidenced by principal component analysis, which collapses the data into a rendering that separates the different treatments (Fig. 7, *A-D*). Specifically, when compared with nontarget siRNA-treated cells, HACD3 silencing causes a depletion of 16:0/18:3, 14:0/18:1, and 16:0/20:3 PCs and an excess of 16:0/16:1 PEs, as can be seen in the hierarchically ordered heat maps (Fig. 7, *E* and *F*). While these changes in phospholipid composition between control and HACD3 siRNA-treated cells are modest compared with the effect of silencing AdipoR2 (Fig. 7, *A*–*F* and Table S2), they are much more pronounced than the changes observed by silencing ALDOC, another conserved PAQR-2/AdipoR2 interactor identified by immunoprecipitation but unlikely to directly influence lipid metabolism (ALDOC is a glycolytic enzyme).

Finally, we used the Laurdan dye method to monitor membrane packing in living cells: this dye emits fluorescence of a different wavelength when present in tightly (more liquid ordered) *versus* loosely (more liquid disordered) packed membranes, and measuring the ratio of these emitted signals yields a global polarization index that correlates with membrane packing (51). Using this method, we found that silencing either ACSL4 or HACD3 in PA-challenged HEK293 cells causes a significant increase in membrane packing, although this effect was not as pronounced as silencing AdipoR2 itself (Fig. 7, *G*–*J*). These results are consistent with the hypothesis that AdipoR2 acts, in part, *via* the ACSL4 and HACD3 proteins to regulate membrane composition.

## Discussion

**Figure 8:**
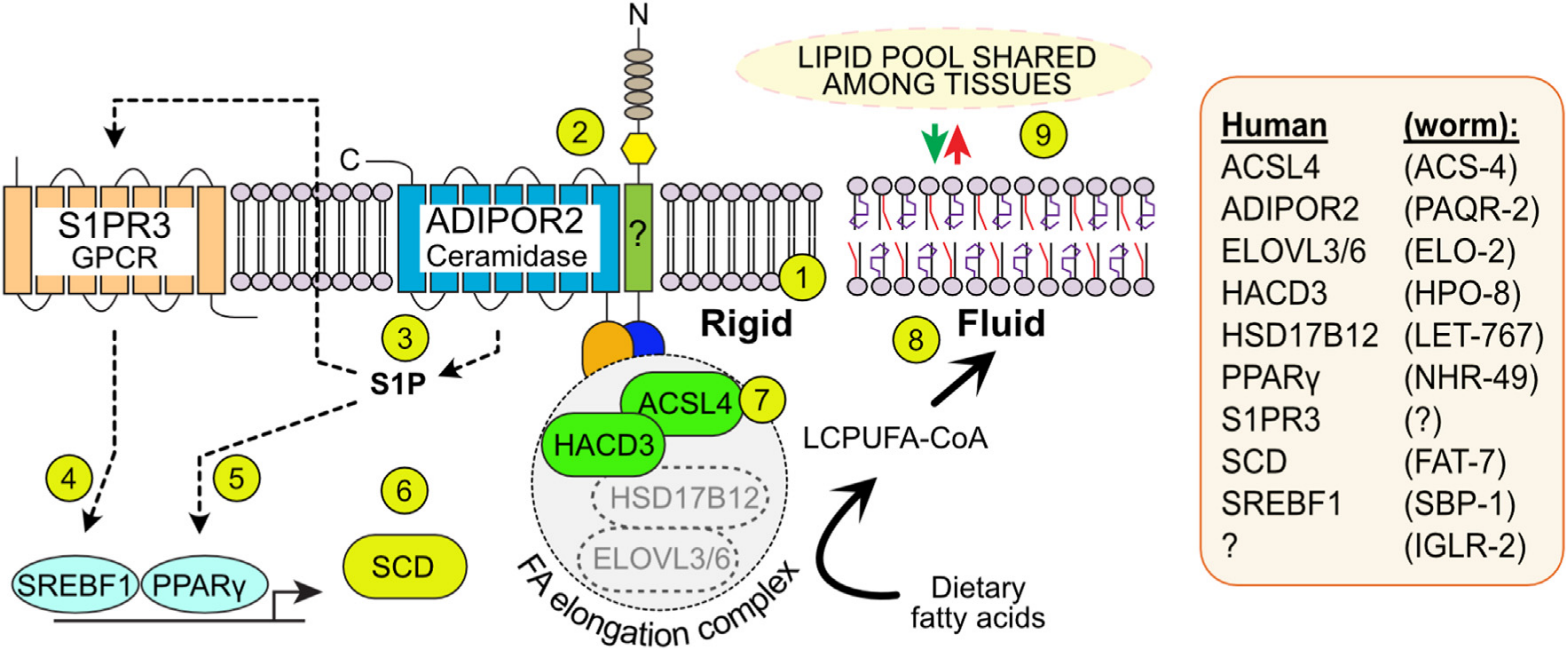
**Model of the AdipoR2 pathway.** AdipoR2 pathway: membrane rigidification ① leads to clustering (with IGLR-2 in *C. elegans*) and activation of the AdipoR2 ceramidase ②, producing sphingosine-1-phosphate (S1P) ③ that activates SREBFs ④ and PPARγ ⑤ leading to desaturase (SCD) expression ⑥. Separately, AdipoR2 recruits a fatty acid elongation complex ⑦ that elongates and desaturates fatty acids then channels them through ACSL4 for incorporation into phospholipids that restore fluidity ⑧ and are shared with other cells ⑨. Worm homologs are listed at *right*.

In this study, we showed that AdipoR2-deficient cells are defective in their ability to further elongate, desaturate, and incorporate LA and its derivates into phospholipids and thus that functional AdipoR2 can promote these processes. We also performed immunoprecipitations of HA::PAQR-2 and of HA::AdipoR2 followed by mass spectrometry and identified several coprecipitated proteins including IGLR-2 in *C. elegans* (no IGLR-2 ortholog has so far been identified in human), many proteins involved in the life cycle of membrane proteins (*e.g.*, ribosomal, coatomer, proteasome proteins), and, most interestingly, several proteins implicated in fatty acid metabolism, including HACD3 (HPO-8 in *C. elegans*) that is part of an ER-bound fatty acid elongase complex (38) and ACSL4 (ACS-4 in *C. elegans*) that is important for UFA activation and their channeling into phospholipids (48). The interactions specifically studied in the present work, namely, PAQR-2/HPO8/ACS-4 (worms) and AdipoR2/HACD3/ACSL4 (human), are experimentally well supported in several ways: (1) detection of the same interactions in two organisms (worms and humans) separated by well over 500 million years (the odds of coincidence are exceedingly low); (2) the specific interactions included three of the four enzymes required for fatty acid elongation (odds of coincidence again exceedingly low); (3) colocalization of the studied interactors in both worms and human cells; (4) validation of the interactions in human cells using BiFC; and (5) the interactions were first discovered using unbiased immunoprecipitations of HA-tagged PAQR-2 or AdipoR2 followed by mass spectroscopy and later confirmed by the separate immunoprecipitations of HA::PAQR-2, MYC::HPO-8, and ACS-4::FLAG in *C. elegans* followed by Western blot detection of the other partners. Based on these findings, we propose that the PAQR-2/AdipoR2 proteins can recruit a complex that locally promotes fatty acid elongation and channels the resulting long-chain UFAs for incorporation, *via* ACS-4/ACSL4, into phospholipids to maintain membrane fluidity (Fig. 8).

Previous genetic and cell biology studies suggest that there are at least two “branches” to the AdipoR2/PAQR-2 downstream pathway that helps restore membrane fluidity (reviewed in (26)). The delineation of the ceramidase–S1P–SREBF1/PPARγ axis provides a detailed understanding of the first branch (32). The second branch of the AdipoR2/PAQR-2 pathway promotes incorporation of PUFAs into phospholipids and was until now exemplified by the *fld-1* and *acs-13* mutant alleles in *C. elegans* or by silencing of their homologs (TLCD1/2 and ACSL1) in mammalian cells (22, 23, 52). The actual molecular nature of the second branch had until now not been characterized, but it was known that only by simultaneously combining mutations from both branches could one achieve complete suppression of the *paqr-2* mutant phenotypes (22, 23). One goal here was therefore to leverage a proteomics approach to better understand how AdipoR2 and PAQR-2 promote the incorporation of PUFAs into phospholipids and the identification of HACD3/HPO-8 and ACSL4/ACS-4 as interaction partners provides useful insights. Stringent criteria were used to establish that AdipoR2/PAQR-2 interacts with HACD3/HPO-8 and ASCL4/ACS-4: the interactions had to be reproducible and specific following immunoprecipitation/mass spectrometry, they were confirmed with follow-up immunoprecipitations/Western blotting of endogenously tagged *C. elegans* proteins, and colocalization was confirmed by detecting tagged proteins in worms and cells, and using BiFC in cells. In addition, silencing the expression of HACD3 or ACSL4 results in membrane composition defects (this work and (50)) accompanied by membrane rigidification in human cells (Fig. 7), and the *C. elegans hpo-8* mutant also has rigid membranes and is cold intolerant (Fig. 6). Altogether, these results are consistent with HACD3/HPO-8 and ACSL4/ACS-4 acting downstream of AdipoR2/PAQR-2 and accounting for the “second branch” in the pathway: promotion of fatty acid elongation and channeling the resulting long-chain UFAs for incorporation into phospholipids to restore plasma membrane fluidity.

Three of four ER-resident enzymes required to form a fatty acid elongation complex were found to interact with AdipoR2/PAQR-2: HACD3 (38), HSD17B12 (also known as KAR; (53)), and ELOVL3 (39, 54) (reviewed in (37)). This suggests either that AdipoR2/PAQR-2 are ER-resident proteins during these interactions or that they are present at the plasma membrane where they recruit an ER-bound complex. Earlier studies showed that AdipoR2 is present both in the ER and the plasma membrane and may have important homeostatic functions in both membranes (29, 33). Importantly, membrane contact sites between the ER and the plasma membrane are well documented and offer a potent mechanism through which ER-bound fatty acid metabolism can be coupled with plasma membrane homeostasis (55, 56). In particular, glycerophospholipids can be delivered directly from the ER to the plasma membrane *via* such contact sites and thus sustain composition homeostasis much faster than *via* vesicular trafficking (57, 58, 59), *i.e.*, the ER–plasma membrane contact sites “provide a nexus for coordinating the complex interrelationship between sterols, sphingolipids, and phospholipids that maintain plasma membrane composition and integrity” (60). Speculatively then, such ER–plasma membrane contact points could allow the evolutionarily conserved interaction between AdipoR2/PAQR-2 and HACD-3/HPO-8-containing fatty acid elongation complex to support membrane homeostasis locally and dynamically in response to dietary or temperature variations.

The different elongases are responsible for elongating fatty acids of various lengths, acting as “molecular calipers” in this process (39, 40). We found that ELO-2 (homologous to human ELOVL3 and ELOVL6) coimmunoprecipitated with HA::PAQR-2 in *C. elegans*. ELO-2 is required for the elongation of PA (16:0) to stearic acid (18:0) and of C18 PUFAs into C20 PUFAs. The mutant therefore accumulates an excess of PA and a depletion of C18 and C20 species within phospholipids, which is accompanied with several defects including poor growth, reduced brood size, increased embryonic/larval lethality, defective rhythmic behaviors, and pale coloring of the intestine (61); many of these phenotypes are also found in *paqr-2* mutants, including the excess PA and depletion of long-chain PUFAs in phospholipids that likely explains the other, secondary physiological phenotypes (62).

That AdipoR2/PAQR-2 interacts with ACSL4/ACS-4 to channel long-chain PUFAs toward phospholipid incorporation, as proposed here, is consistent with two large-scale unbiased CRISPR/Cas9 screens previously performed in human cells. In a gene inactivation screen, both AdipoR2 and ACSL4 ranked among the most essential genes for tolerance to exogenously supplied SFAs (48). Conversely, in a gene activation screen, AdipoR2 and ACSL4 ranked among the genes that best conferred SFA tolerance when overexpressed (63).

Others have previously sought to identify AdipoR interactors. In particular, a yeast two-hybrid screen using the seventh transmembrane domain of AdipoR1 as bait identified APPL1 as a putative functional interactor (64). Also, a single AdipoR homolog exists in *Drosophila melanogaster*, dAdipoR, and immunoprecipitation of a MYC-tagged dAdipoR followed by mass spectrometry identified several potential interactors, including OSTΔ and Stt3B (both involved in protein glycosylation) and HSC70 (ER resident chaperone) that likely contribute to the dAdipoR protein life cycle (65). No members of the elongase complex or ACSL were identified in either of these studies, which may reflect differences in the methods used.

In the future, it will be interesting to investigate the roles of the other candidate interactors in AdipoR2/PAQR-2-mediated membrane homeostasis, to test whether these interactions are regulated by membrane properties and whether they occur sequentially or simultaneously in the form of a large complex.

## Experimental procedures

### *C. elegans* strains and cultivation

The wildtype *C. elegans* reference strain N2, the *hpo-8* mutant strain VC2093 (*T15B7.2(ok2680) V/nT1 [qls51]*), and the *acs-4* mutant strain VC2240 (*acs-4(ok2872) III/hT2 [bli-4(e937) let-?(q782) qIs48]*) are available from the *C. elegans* Genetics Center. Unless otherwise stated, the *C. elegans* strains maintenance and experiments were performed at 20 °C using the *E. coli* strain OP50 as food source, which was maintained on LB plates kept at 4 °C (restreaked every 6–8 weeks) from which single colonies were picked for overnight cultivation at 37 °C in LB medium, then used for seeding nematode growth medium (NGM) plates. OP50 stocks were kept frozen at −80 °C and new LB plates were streaked every 3 to 4 months. Filter sterilized glucose was added to the molten medium to prepare NGM plates containing 20 mM glucose.

The *paqr-2(syb364)* allele in which a HA tag was inserted at the beginning of the *paqr-2* locus has been described (25); briefly, the genomic locus of *paqr-2* was modified by introducing the HA-tag sequence fused in frame to the START codon of *paqr-2*.

The *acs-4(syb1478)* allele in which a 3XFLAG tag was fused in frame to the end of the *acs-4* locus, just upstream of the STOP codon, was created by Suny Biotech Co using CRISPR/Cas9. The altered sequence is as follows (3XFLAG tag coding sequence is in upper case; endogenous *acs-4* sequence is in lower case, with STOP codon underlined): 5′-gcactcaagctgaagagaaagcctatccaaatggcctaccagaagaccctggatgatctgtataagcagctcaaaaagaatGATTACAAAGACCATGACGGTGACTATAAGGATCACGATATCGATTACAAAGACGATGACGACAAGtgattcatgtcttcctagctttctttttttcttttcccgcccttttcacactggtaacttgctcttccccacctattcgagata-3′.

The *hpo-8(syb2306)* allele in which a MYC tag was fused in frame to the START codon of *hpo-8* was created by Suny Biotech Co using CRISPR/Cas9. The altered sequence is as follows (MYC tag coding sequence is in upper case; endogenous *hpo-8* sequence is in lower case, with START codon underlined): 5′-cgtgtggcgtgatatacttttctcattttcttttctttgcattgcctaaaatccttatttttgcagggtaacaatgGAACAAAAGCTTATCTCAGAAGAAGATCTGagcgttcagacctatctggttgcgtacaacgtgttacaaattttagggtacgtttttttttctgaaagaagttcac-3′.

### HEK293 cell maintenance

HEK293 cells were obtained from the American Type Culture Collection and grown in Dulbecco’s modified Eagle’s medium containing glucose 1 g/l, pyruvate, and GlutaMAX and supplemented with 10% fetal bovine serum, 1% nonessential amino acids, Hepes 10 mM, and 1% penicillin and streptomycin (all from Life Technologies) at 37 °C in a water-humidified 5% CO2 incubator. Cells were subcultured twice a week at 90% confluence. TrypLE Express reagent (Gibco) was used to detach the cells when subculturing. HEK293 were cultivated on treated plastic flasks and multidish plates (Nunc). For confocal microscopy cells were seeded on Ibidi glass bottom μ-slides or μ-dishes.

### Immunoprecipitations from *C. elegans* and HEK293 cells

Worms from five 10-cm plates containing crowded but not starved worms were collected using M9 buffer and transferred to 10-ml tubes, then washed three times using M9, and centrifuged at 850*g*. Worms were then incubated for 20 min on ice in 300 μl of 25 mM Tris (pH-7.5), 300 mM NaCl, 0.1% NP-40, and 1X protease inhibitor (the protease inhibitor was excluded for samples later analyzed using mass spectroscopy). The worms were disrupted using a motorized pestle while kept on ice, centrifuged at 20,000*g* in a microfuge at 4 °C for 20 min, and the supernatant was transferred to a new tube. Rabbit monoclonal anti-HA antibody, 7 μl (C29F4, Cell signaling, RRID: AB_1549585, Cat# 3724) and 20 μl of protein A-agarose beads (Roche) were added to the lysate, which was then rotated at 4 °C for 1 h (samples for mass spectroscopy). The beads were then washed three times with lysis buffer (without protease inhibitor). For mass spectroscopy analysis, bound proteins were then eluted in 50 μl of 0.2 M glycine pH 2.5, then neutralized with 1 M Tris pH 8.0.

For the FLAG immunoprecipitation (IP), worms were lysed in 10 mM Tris (pH 7.5), 150 mM NaCl, 5 mM EDTA, 0.5% NP40, and 1× protease inhibitor on ice with a motorized pestle. The protein lysate, 1000 μg, was added to 40 μl anti-FLAG M2 magnetic beads (Sigma-Aldrich, Cat# M8823) and rotated at 4 °C overnight. The beads were magnetically separated and washed three times in 10 mM Tris (pH 7.5), 150 mM NaCl, 5 mM EDTA. Bound proteins were eluted with 50 μl 0.2 M glycine (pH 2.5) and neutralized with 1 M TEAB, 1× loading buffer containing β-mercaptoethanol was added to the eluate, and samples were boiled for 10 min at 95 °C before loading on a gel.

For the MYC IP, worms were lysed in the Next Advance Bullet Blender Homogenizer in lysis buffer containing 10 mM Tris (pH 7.5), 150 mM NaCl, 5 mM EDTA, 0.5% NP40, and 1× protease inhibitor with 0.2-mm stainless steel beads for 3 min at 4 °C. The protein lysate, 800 μg, was added to 25 μl Myc-Trap magnetic agarose beads (Chromotek, Cat# ytma-10) and rotated for 75 min at 4 °C. Beads were magnetically separated and washed three times in wash buffer containing 10 mM Tris (pH 7.5), 150 mM NaCl, 5 mM EDTA. Proteins were eluted from the beads using 35 μl 2× loading buffer containing β-mercaptoethanol and boiled at 95 °C for 10 min.

For the HA IP, worms were lysed in 25 mM Tris (pH 7.5), 150 mM NaCl, 0.1% NP40, and 1× protease inhibitor on ice with a motorized pestle. The lysate, 1000 μg, was added to 25 μl HA-Tag (C29F4) rabbit mAb Sepharose bead conjugate (Cell signaling, Cat# 3956) and rotated at 4 °C overnight. The beads were separated and washed three times in wash buffer containing 10 mM Tris (pH 7.5), 150 mM NaCl, 5 mM EDTA. Bound proteins were eluted in 40 μl 2× loading buffer containing β-mercaptoethanol, and samples were boiled for 10 min at 95 °C before loading on a gel.

HEK293 cells stably expressing AdipoR2-HA (34) were lysed in 25 mM Tris (pH 7.5), 300 mM NaCl, 0.1% NP-40 buffer and incubated with protein A-agarose beads (Roche) and rabbit monoclonal anti-HA antibody (C29F4, Cell signaling, RRID: AB_1549585, Cat# 3724) for 3 h, washed with lysis buffer three times, eluted with 0.2 M Glycine pH 2.5, and neutralized the elute with Tris pH 8.0.

### Proteomic analysis and protein identification

Proteomic analysis was performed at The Proteomics Core Facility at Sahlgrenska Academy, Gothenburg University. The *C. elegans* samples were digested using the filter-aided sample preparation method (66). The HEK293 samples were digested using the suspension trapping (S-Trap, Protifi) spin column digestion method according to the manufacturer instructions. All samples were desalted using PepClean C18 spin columns (Thermo Scientific) according to the manufacturer’s guidelines prior to analysis on a QExactive HF or a Orbitrap Fusion Tribrid mass spectrometer interfaced with Easy nLC 1200 liquid chromatography system (Thermo Scientific). Peptides were trapped on an Acclaim Pepmap 100 C18 trap column (100 μm × 2 cm, particle size 5 μm, Thermo Scientific) and separated on an in-house packed analytical column (75 μm × 30 cm, particle size 3 μm, Reprosil-Pur C18, Dr Maisch). The instruments operated in data-dependent mode where the precursor ion mass spectra were acquired at a resolution of 60K and 120K, respectively. The most intense multiply charged ions were selected for fragmentation, for QEHF collision energy HCD settings at 28 and MS2 spectra recorded at a resolution of 30K and for Fusion collision energy CID setting at 35 with detection in the ion trap. Data analysis was performed utilizing Proteome Discoverer version 1.4 (Thermo Scientific) against SwissProt *C. elegans* (Nov 2017, 3972 sequences) and UniProt *C. elegans* (Sep 2017, 26,778 sequences). HEK293 samples were matched against SwissProt Human (June 2019, 20,432 sequences) or against a custom database containing the AdipoR2 sequence including tag. Mascot 2.5 (Matrix Science) was used as a search engine with precursor mass tolerance of 5 ppm and fragment mass tolerance of 200 mmu for *C. elegans* and 30 mmu for Human raw data. Tryptic peptides were accepted with one missed cleavage. Methionine oxidation was set as variable modifications and cysteine alkylation as static. The filter for PSM validation and identified peptide in the software was set at 1% false discovery rate by searching a reversed database, and identified proteins were grouped when sharing the same sequences to minimize redundancy. The primary proteomics data are available *via* ProteomeXchange (http://www.proteomexchange.org) with identifier PXD031395.

### *C. elegans* dietary *E. coli* preloading with fatty acids

Bacteria were loaded with fatty acids as described (20). Briefly, the fatty acids were added to LB cultures of OP50 bacteria, grown overnight at 37 °C, and washed to remove LB and fatty acids in solution. Then, the fatty acid–loaded OP50 bacteria were seeded onto NGM plates lacking peptone.

### *C. elegans* growth assay

For length measurement studies, synchronized L1s were plated onto test plates seeded with *E. coli* and worms were mounted and photographed 144 h (experiments at 15 °C) or 72 h (all other experiments) later. The length of 20 worms was measured using ImageJ.

### *C. elegans* plasmids and injections

The *pPAQR-2::N-GFP* construct has been described elsewhere (67). The *pACS-4::mCherry* construct was generated using Gibson-assembly cloning kit (NEB) with the following primers: 5′-ctcaaaaagaatatggtgagcaagggcgaggaggataaca-3′ and 5′-tggaattctacgaatgctacttgtacagctcgtccatgcc-3′ for amplification of the mCherry fragment using the *pIGLR-2::mCherry* construct (30) as a template and 5′-tagcattcgtagaattccaactgagcgccggtcgctaccatt-3′ and 5′-caccatattctttttgagctgcttatacagatcatcgaggg-3′ for amplification of the *acs-4* fragment and rest of the vector sequence using the *pACS-4::GFP* (68) construct as a template. The *pACS-4::GFP* construct was a kind gift from Prof. Jennifer Watts. The combination of plasmids *pPAQR-2::N-GFP* and *pACS-4::mCherry* was coinjected into N2 worms with a concentration of 25 ng/μl for each of the plasmid. The *pHPO-8::mCherry* construct was generated using Gibson-assembly cloning kit (NEB) with the following primers: 5′-gaagaagcagatggtgagcaagggcgaggaggataacatg-3′ and 5′-gaatttttctctacttgtacagctcgtccatgccgccggt-3′ for amplification of the mCherry fragment using the *pACS-4::mCherry* construct as a template, 5′-ccttgtctagatttttcctatttcttgcgtcaattcgg-3′ and 5′-gctcaccatctgcttcttcttgcttcctccgccgagaa-3′ for amplification of the *hpo-8* fragment and 5′-gctgtacaagtagagaaaaattctacgtgtatttatattacag-3′ and 5′- ttcgcccttacaaattctgcagaaaagcatttccattg-3′ for amplification of 3′-UTR using N2 genomic DNA, 5′-cagaatttgtaagggcgaattctgcagatatccatcacac-3′ and 5′-ataggaaaaatctagacaagggcgaattccagcacactgg-3′ for amplification of the vector sequence using the *pPAQR-2::N-GFP* construct as a template. The combination of plasmids *pPAQR-2::N-GFP* and *pHPO-8::mCherry* was coinjected into N2 worms at a concentration of 25 ng/μl and 1 ng/μl of each plasmid, respectively.

### *C. elegans* colocalization experiments

N2 worms expressing GFP::PAQR-2 and ACS-4::mCherry or GFP::PAQR-2 and HPO-8::mCherry were bleach-synchronized and spotted on NGM plates. After 72 h, day 1 adults were mounted on agarose slides and images were acquired using a Zeiss LSM700inv confocal microscope with a 40× water immersion objective. Pearson correlation coefficient was calculated in a region of interest using the Coloc2 plugin in Fiji/ImageJ.

### FRAP experiments in *C. elegans*

FRAP experiments in *C. elegans* were carried out using a membrane-associated prenylated GFP reporter expressed in intestinal cells as described (69) and using a Zeiss LSM700inv laser scanning confocal microscope with a 40× water immersion objective. Briefly, the GFP-positive membranes were photobleached over a rectangular area (30 × 4 -pixels) using 30 iterations of the 488-nm laser with 50% laser power transmission. Images were collected at a 12-bit intensity resolution over 256 × 256 pixels (digital zoom 4×) using a pixel dwell time of 1.58 μs and were all acquired under identical settings. The recovery of fluorescence was traced for 25 seconds. Fluorescence recovery and *T*_half_ were calculated as described (19).

### HEK293 fatty acid treatment

Palmitic acid (PA; 16:0) was dissolved in sterile dimethyl sulfoxide then mixed with fatty acid–free bovine serum albumin (BSA) (all from Sigma) in serum-free medium for 15 min at room temperature. The molecular ratio of BSA to fatty acid was 1 to 2.65. Cells were then cultivated in this serum-free medium containing PA for 24 h prior to analysis. 13C-labeled OA and 13C-labeled LA were obtained from Larodan as solution in ethanol and conjugated to BSA as described above. HEK293 in serum-free medium were treated with 13C-labeled OA or13C-labeled LA at 25 μM for 6 h.

### HEK293 siRNA treatment

The following predesigned siRNAs were purchased from Dharmacon: ACSL4 J-009364-05, AdipoR2 J-007801-10, AldoC siRNA J-012697-07, HACD3 J-010664-09, and Non-Target (NT) D-001810-10. HEK293 cell transfection was performed in complete media using 25 nM siRNA and Viromer Blue according to the manufacturer’s instructions 1× (Lipocalyx). The Viromer Blue reagent was chosen for siRNA delivery because it relies on viral-like proteins rather than traditional liposomes that could affect the membrane composition of the target cells. Knockdown gene expression was verified 48 h after transfection (Fig. S1*B*).

### HEK293 quantitative PCR

Total cellular RNA was isolated using RNeasy Plus Kit according to the manufacturer’s instructions (Qiagen) and quantified using a NanoDrop spectrophotometer (ND-1000; Thermo Fisher). cDNA was obtained using a RevertAid H Minus First Strand cDNA Synthesis Kit with random hexamers (Thermo Fisher). qPCR experiments were performed with a CFX Connect thermal cycler (Bio-Rad) using Hot FIREpol EvaGreen qPCR SuperMix (Solis Biodyne) and standard primers. The relative expression of each gene was calculated according to the ΔΔCT method (70). Expression of the housekeeping gene PPIA was used to normalize for variations in RNA input. Primers designed for this study were ALDOC-For AATGGTGTTCCCTTCGTCCG, ALDOC-Rev AGCCCTTGAGTGGTGGTTTC, HACD3-For GAAAGCGAAGGCTCTCCTGA, and HACD3-Rev TTTCCACAACTGCCAGCATC. Primer sequences for ACSL4, AdipoR2, and PPIA were described in (23, 24).

### HEK293 plasmids, transfection, and bimolecular fluorescence complementation

The pIREShyg2-HA-hAdipoR2-cMYC construct was described in (25). The ACSL4_v1-FLAG construct was described in (71) and was a kind gift from Prof. Joachim Fullekrug. Plasmid HsCD00674547 expressing HACD3/PTPLAD1 was obtained from DNASU Plasmid Repository, Arizona State University. The pIREShyg2-FLAG-HACD3 construct was generated using Gibson-assembly cloning kit (NEB) with the following primers: 5′-ctacaaggacgacgatgacaaggagaatcaggtgttgacgcc-3′ and 5′-aatccggatcagtggatctttttctttttttgtccatagcgc-3′ for amplification of the HACD3 fragment; 5′-gatccactgatccggattcgaattcggatccgcggccgca-3′ and 5′-tcatcgtcgtccttgtagtccatggtggctagctaggccg-3′ for amplification of the vector sequence. HEK293 cells were transfected using Viromer Red according to the manufacturer’s instructions 1× protocol (Lipocalyx), and the protein expression was verified by Western blot 24 h after transfection.

The pCE-IGLR2-VC155 and pCE-VN173-PAQR2 plasmids were used from a previously published paper (19) for amplification of C-terminal and N-terminal Venus fluorophore. The pIREShyg2-VN173-HA-hAdipoR2-cMYC construct was generated using Gibson-assembly cloning kit (NEB) with the following primers: 5′-gcatcaatggtgagcaagggcgaggagctgttcaccgggg-3′ and 5′-tctgggacgtcgtatgggtactcgatgttgtggcggatcttg-3′ for amplification of the VN173 fragment; 5′-tacccatacgacgtcccagactacgctaacgagccaacag-3′ and 5′-cccttgctcaccattgatgccatggtggctagcgcgccgg-3′ for amplification of HA-hAdipoR2-cMYC and rest of the vector sequence.

The pIREShyg2-hACSL4_v1-FLAG-VC155 construct was generated using Gibson-assembly cloning kit (NEB) with the following primers: 5′-gctagccaccatggcaaagagaataaaagctaagcccact-3′ and 5′-ctttgtagtctttgcccccatacattcgttcaatgtctttga-3′ for amplification of the ACSL4_v1 fragment; 5′-tgggggcaaagactacaaagacgatgacgacaaggacaag-3′ and 5′-tttgccatggtggctagcgcgccggcttaaggcctgtac-3′ for amplification of the VC155 fragment and rest of the vector sequence.

The pIREShyg2-FLAG-hHACD3-VC155 construct was generated using Gibson-assembly cloning kit (NEB) with the following primers: 5′-gctagccaccatggagaatcaggtgttgacgccgcatgtc-3′ and 5′-ctttgtagtcgtggatctttttctttttttgtccatagcg-3′ for amplification of the HACD3 fragment; 5′-aaagatccacgactacaaagacgatgacgacaaggacaagca-3′ and 5′-gattctccatggtggctagcgcgccggcttaaggcctgta-3′ for amplification of the VC155 fragment and rest of the vector sequence.

The different combinations of BiFC plasmids were transfected in HEK293 cells using Viromer Red according to the manufacturer’s instructions 1× protocol (Lipocalyx). Twenty-four hours post transfection, images were acquired with an LSM880 confocal microscope equipped with a live cell chamber (set at 37 °C and 5% CO2) using a 40× water-immersion objective. Cells were excited with a 488-nm laser and the emission between 493 and 589 nm was recorded.

### Protein extraction and Western blotting

Protein, 20 or 50 μg, HEK293 samples or worm IP input samples, respectively, was mixed with Laemmli sample loading buffer (Bio-Rad), heated to 37 °C (HEK293) or 95 °C (worm samples) for 10 min, and loaded in 4% to 20% gradient precast SDS gels (Bio-Rad). After electrophoresis, the proteins were transferred to nitrocellulose membranes using Trans-Blot Turbo Transfer Packs and a Trans-Blot Turbo apparatus/predefined mixed-MW program (Bio-Rad). Blots were blocked with 5% nonfat dry milk in PBS-T for 1 h at room temperature. Blots were incubated with primary antibodies overnight at 4 °C: rabbit monoclonal anti-HA antibody (C29F4, Cell signaling, RRID: AB_1549585, Cat# 3724) 1:5000 dilution, mouse monoclonal anti-FLAG antibody (M2, Sigma-Aldrich, RRID: AB_259529, Cat# F3165) 1:3000 dilution, mouse monoclonal anti-alpha-Tubulin (B512, Sigma-Aldrich, RRID: AB_477579, Cat# T5168) 1:5000 dilution, or mouse monoclonal anti-Myc antibody (My3, MBL, RRID: AB_11161202, Cat# M192-3MS) 1:1000 dilution. Blots were then washed with PBS-T and incubated with swine anti-rabbit HRP (Dako, RRID: AB_2617141, Cat# P0399) 1:3000 dilution, goat anti-mouse HRP (Dako, RRID_ AB_2617137) 1:3000 dilution, or TidyBlot Western blot detection HRP (Bio-Rad, Cat# STAR209) 1:400 dilution and washed again with PBS-T. Detection of the hybridized antibody was performed using an ECL detection kit (Immobilon Western, Millipore), and the signal was visualized with a digital camera (VersaDoc, Bio-Rad).

### HEK293 immunofluorescence

HEK293 cells were fixed with 4% formaldehyde for 20 min at room temperature. Fixed cells were then washed with PBS and permeabilized with 0.1% saponin and blocked with 0.5% BSA and 0.5% gelatin for 60 min. Cells were washed once with washing buffer (0.01% saponin, 0.2% gelatin in PBS) and incubated with mouse monoclonal anti-FLAG M2 antibody (Sigma) and rabbit monoclonal anti-HA antibody (C29F4, Cell Signaling) for 60 min. Cells were then washed with washing buffer and incubated with secondary antibodies, anti-mouse Alexa 568 (Invitrogen) and anti-rabbit Alexa 488 (Invitrogen) for 60 min. After washing with washing buffer, mounting media with NucBlue (Invitrogen) was added to stain the nuclei. Images were acquired with an LSM880 confocal microscope with a 40× water-immersion objective. Pearson correlation coefficient was calculated with ImageJ, as described (23).

### Measurement of membrane order

Live HEK293 cells were stained with Laurdan dye (6- dodecanoyl-2-dimethylaminonaphthalene) (Thermo Fisher) at 15 μM for 45 min and imaged using an LSM880 confocal microscopy equipped with live cell chamber (set at 37 °C and 5% CO2) as described (24). Images were analyzed using ImageJ version 1.47 software, following published guidelines (51).

### Lipidomics of nonlabeled samples

Cell preparations (in at least three independent replicates) and lipid extraction and analysis were performed as in (32). Briefly, phospholipid composition was determined by direct infusion (shotgun approach) into a QTRAP 5500 mass spectrometer (Sciex) equipped with a Triversa nanomate (Advion Bioscience) as described (72). For PCs, mass spectra were attained in precursor ion scanning mode using the phosphocholine headgroup *m/z* 184.1 as fragment ion. For PEs, neutral loss scanning of *m/z* 141.1 was used (73, 74). The data were evaluated using the Lipidview and Multiquant software (Sciex). Qlucore omics explorer software was used for the multivariant analysis. The complete lipid composition data are included in Table S2.

### Lipidomics of samples with 13C-labeled substrates

Cellular total lipids were extracted according to Folch *et al.* (75) and dissolved in chloroform/methanol 1:2 (by vol). An internal standard mixture containing representatives for all lipid classes analyzed was added and the quantification of lipids was carried out using a liquid chromatography–tandem mass spectrometry (MS/MS) approach. Chromatographic separation was done in a gradient mode using an Agilent 1290 Infinity HPLC system equipped with a Luna Omega C18 100 Å (50 × 2.1 mm, 1.6 μm) column (Phenomenex) and employing an acetonitrile/water/isopropanol-based solvent system (76). Solvent A was acetonitrile/water (60:40) containing 10 mM ammonium formate and 0.1% formic acid, and solvent B was isopropanol/acetonitrile (90:10) containing 10 mM ammonium formate and 0.1% formic acid. The gradient started from 32% (v/v) B and was maintained for 1.5 min, then reached 45% at 4 min, 52% at 5 min, 58% at 8 min, 66% at 11 min, 70% at 14 min, 75% at 18 min, and 97% at 21 min and was kept there for 4 min, after which the gradient was switched back to 32% in 1 min and kept there for 6 min. The flow rate was 0.2 ml/min and column temperature 25 °C. The column eluent was infused into the electrospray source of an Agilent 6490 Triple Quad LC/MS with iFunnel Technology (Agilent Technologies), and spectra were recorded using both positive and negative ionization modes. Phospholipids were detected using head group–specific precursor ion (P) or neutral loss (NL) scans (PC: P184, PE: NL141). The identity of labeled species was confirmed by direct infusion electrospray ionization–MS/MS (Agilent 6410 Triple Quad) using a flow of 10 μl/min and negative mode precursor ion scans for the labeled acyl chains released from PE, and from PC chloride adducts [M + Cl]^−^. In addition, possible overlaps of the signals from the labeled lipid species and unlabeled species were inspected by recording the negative mode fatty acid specific precursor ion scans, which were compared with the spectra of all other mass spectrometry and MS/MS scans of the labeled and unlabeled samples. Mass spectra were processed using MassHunter Qualitative Navigator software (Agilent Technologies), and lipid species were quantified utilizing the internal standards and LIMSA software (77).

## Data availability

The primary proteomics data are available *via* ProteomeXchange (http://www.proteomexchange.org) with identifier PXD031395. Other data are contained within the article or supporting information.

## Supporting information

This article contains supporting information.

## Conflict of interest

The authors declare that they have no conflicts of interest with the contents of this article.
