# Supplementary material for: AdipoR2 recruits protein interactors to promote fatty acid elongation and membrane fluidity
Source: J Biol Chem. 2023 May 8;299(6):104799. doi: 10.1016/j.jbc.2023.104799 (PMC10279913; doi:10.1016/j.jbc.2023.104799)
Supplement: Supplemental Figures S1–S6 [file mmc3.pdf]

SUPPORTING INFORMATION

TITLE: ADIPOR2 RECRUITS PROTEIN INTERACTORS TO PROMOTE FATTY ACID ELONGATION AND MEMBRANE FLUIDITY

AUTHORS: Mario Ruiz*[1], Ranjan Devkota*[1], Delaney Kaper*[1], Hanna Ruhanen[2,3], Kiran Busayavalasa[1], Uroš Radović[1], Marcus Henricsson[4], Reijo Käkelä[2,3], Jan Borén[4] and Marc Pilon[1#]

*These authors contributed equally.

Affiliations: [1]Department of Chemistry and Molecular Biology, University of Gothenburg, Gothenburg, Sweden, [2]Helsinki University Lipidomics Unit, Helsinki Institute of Life Science, Biocenter Finland, Helsinki, Finland, [3]Molecular and Integrative Biosciences Research Programme, Faculty of Biological and Environmental Sciences, University of Helsinki, Helsinki, Finland [4]Department of Molecular and Clinical Medicine/Wallenberg Laboratory, Institute of Medicine, University of Gothenburg, Gothenburg, Sweden.

CONTENT:

Supplementary FIG. S1

Supplementary FIG. S2

Supplementary FIG. S3

Supplementary FIG. S4

Supplementary FIG. S5

Supplementary FIG. S6

FIG. S1

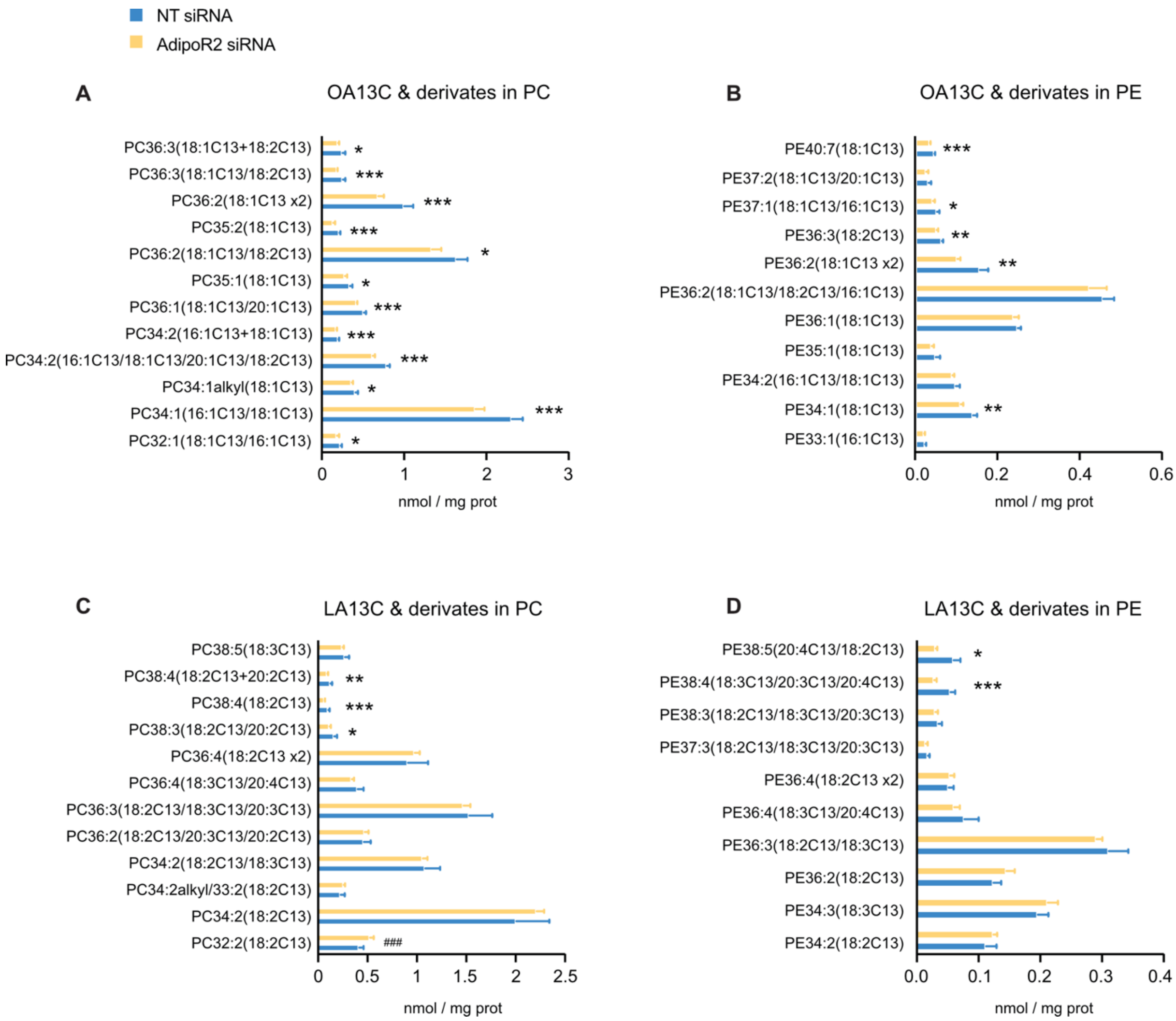

Suppl. Fig. S1. Abundance of labeled fatty acids and their derivates in phospholipids of HEK293 cells. (A-B) AdipoR2 siRNA-treated cells show similarly reduced levels of incorporated 13C-labelled OA (OA13C) and its derivates across all types (length and degree of unsaturation) of PCs and PEs. **(C-D)** AdipoR2 siRNA-treated cells show excess levels of incorporated 13C-labelled LA (LA13C) and its derivates mostly in short, less desaturated PCs and PEs. 13C-labelled fatty acid(s) found in the isobaric lipid species are marked in parenthesis (/ = alternatives, + = both acyl chains were labelled, x2 = two similar labelled acyl chains). * and # indicate significant increase or decrease in AdipoR2 siRNA-treated cells, respectively and using *t*-tests where *p<0.05; ***p*<0.01; and ***/### p<0.001.

FIG. S2

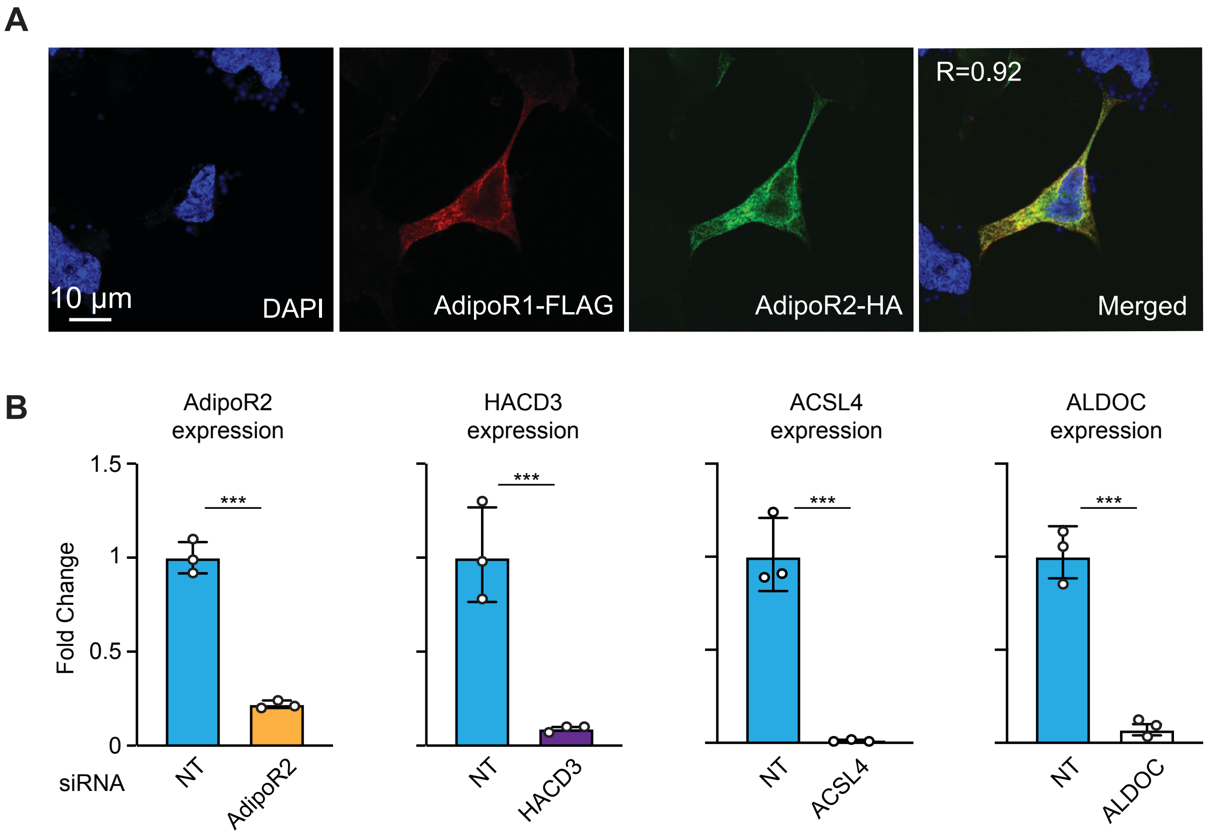

Suppl. Fig. S2. Immunofluorescence of AdipoR1 and AdipoR2 and validation of siRNA silencing. (A) Immunofluorescence imaging confirms that AdipoR1 and AdipoR2 co-localize; R is the Pearson correlation coefficient between the two fluorophores. **(B)** qPCR showing that the four siRNAs used in this study efficiently silenced the targeted genes.

FIG. S3

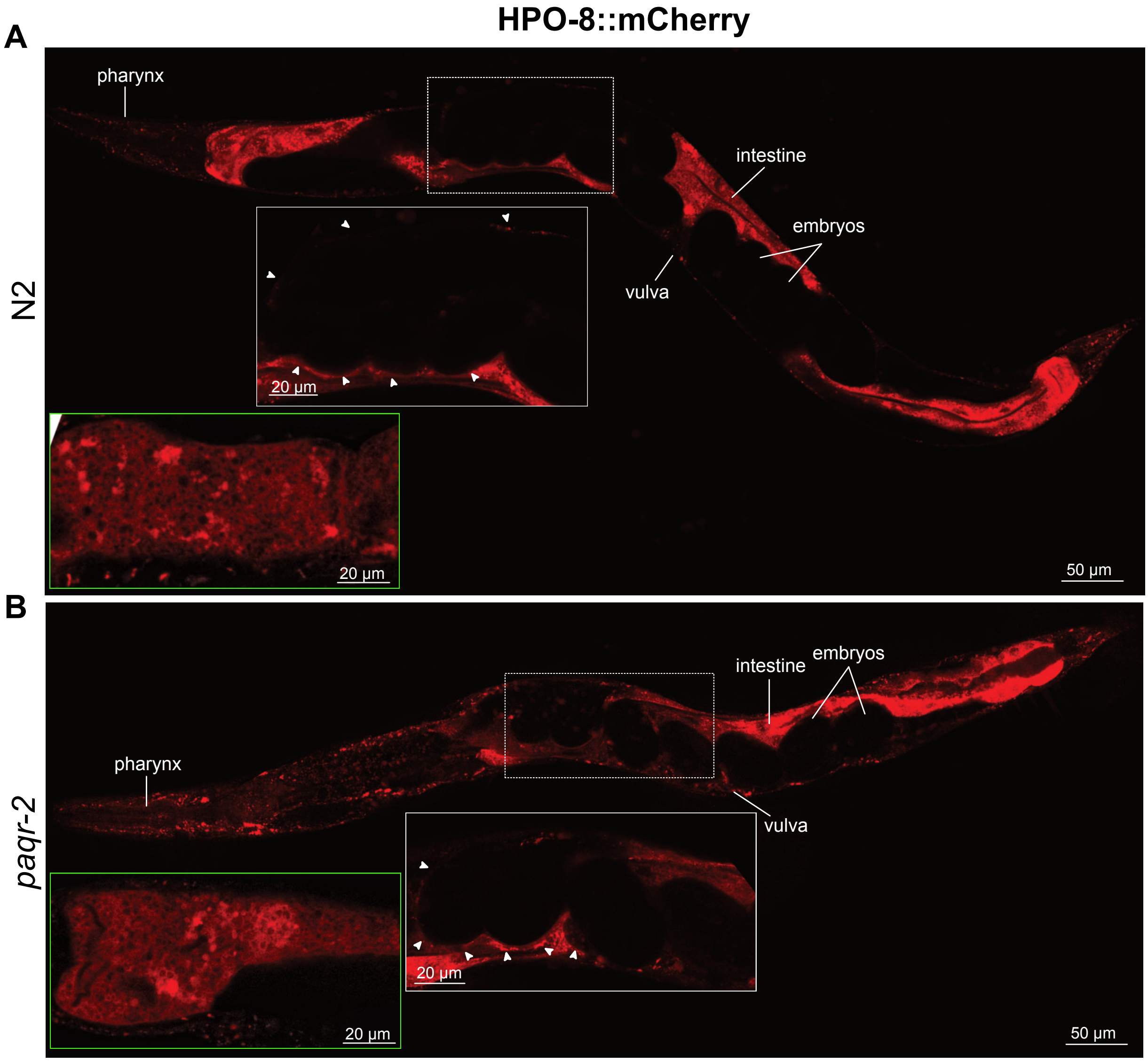

Suppl. Fig. S3. PAQR-2 is not required for HPO-8 Expression and localization. Expression pattern of a HPO-8::mCherry reporter in 1-day old adult wild-type N2 worms (A) and *paqr-2* mutant worms (B). The white-framed inset shows expression in the gonad sheath cells (arrowheads). The green-framed insets are from different worms and show a similar reticular pattern within the first intestinal cells in N2 and *paqr-2* mutant worms.

FIG. S4

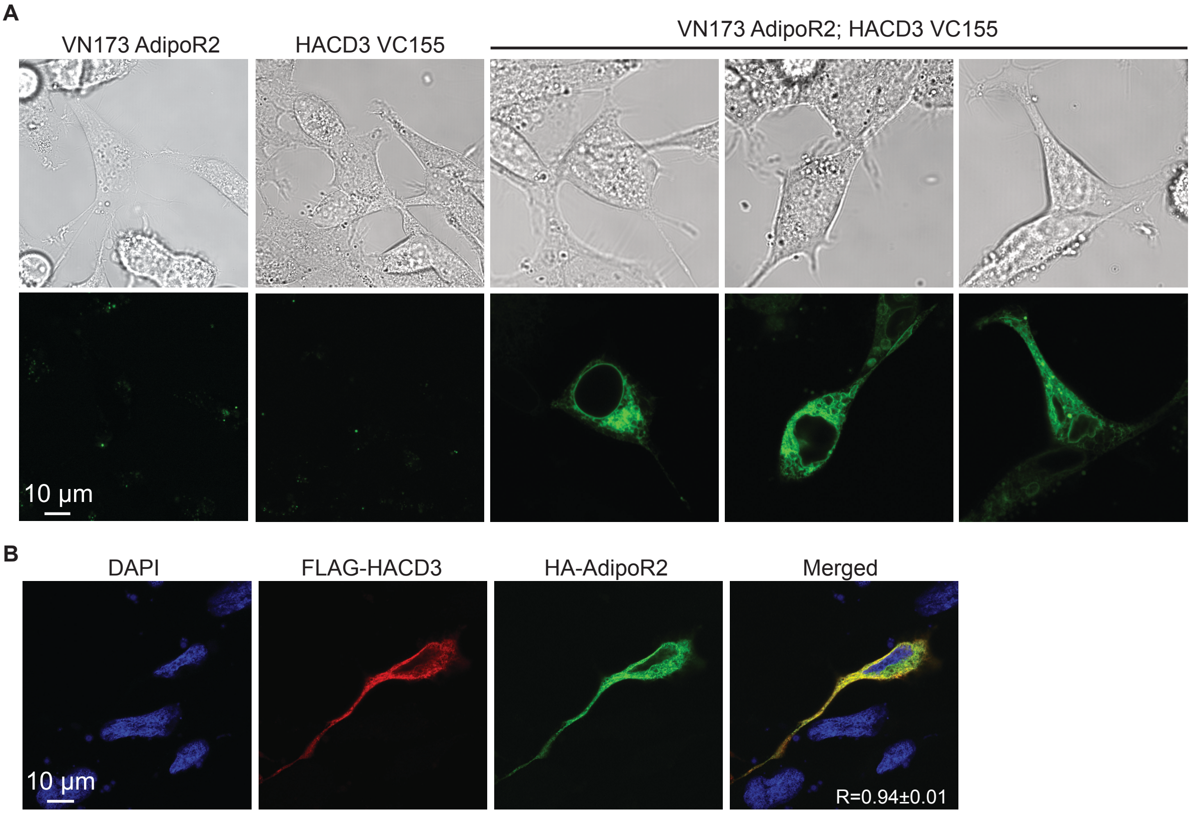

Suppl. Fig. S4. AdipoR2 interacts with HACD3. (A) BiFC experiment confirming that AdipoR2 interacts with HACD3, with three additional examples of reporter co-expression. .Note that the VN173 AdipoR2 panels are the same as in Fig. 3D. (B) Immunofluorescence confirming that HA-AdipoR2 co-localizes with FLAG-HACD3. Note the strong colocalization as indicated by yellow hues in the merged panel. R is the Pearson correlation coefficient between the two fluorophores ± standard deviation (n=5).

FIG. S5

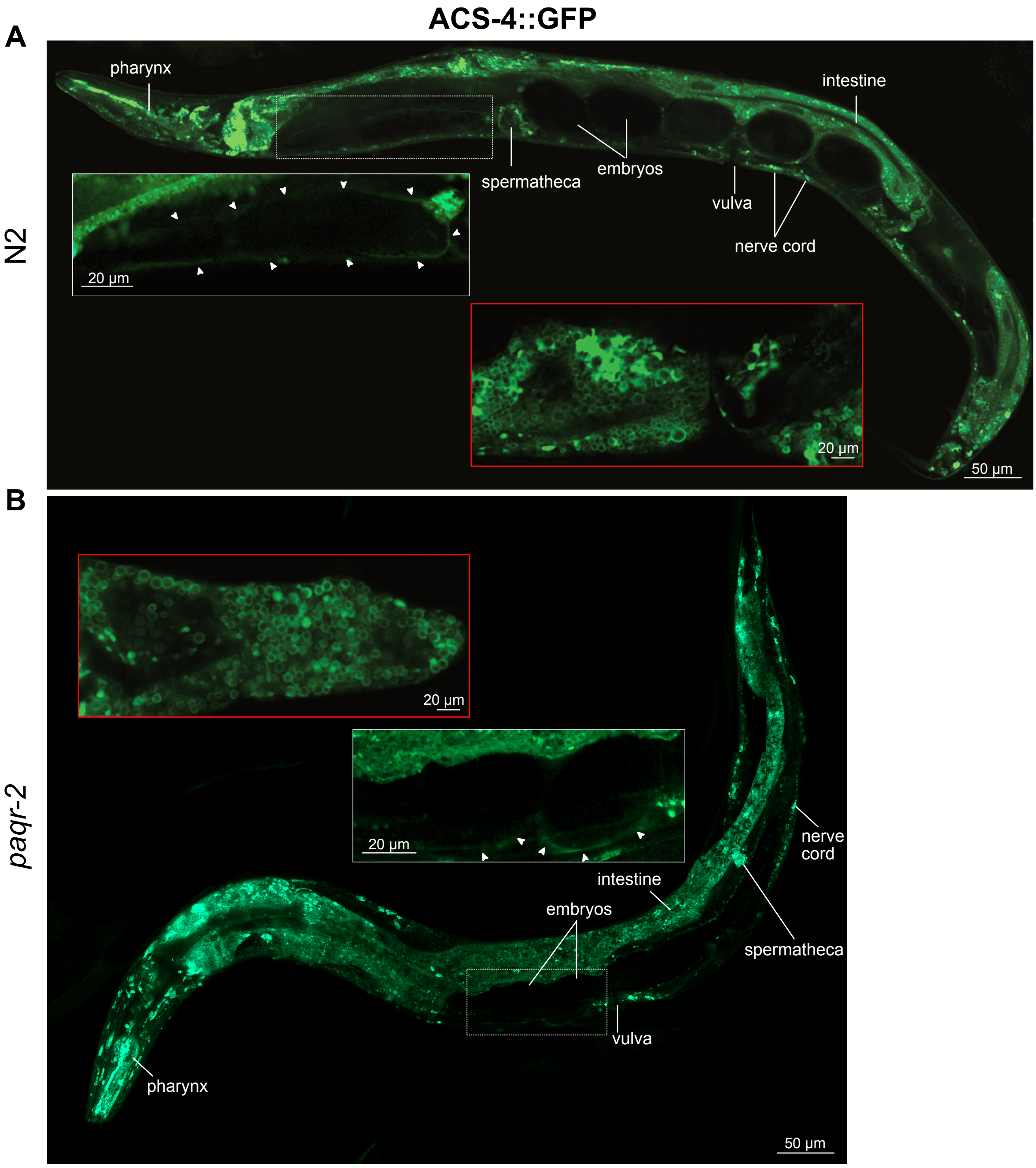

Suppl. Fig. S5. PAQR-2 is not required for ACS-4 Expression and localization. Expression pattern of a ACS-4::GFP reporter in 1-day old adult wild-type N2 worms (A) and *paqr-2* mutant worms (B). The white-framed inset shows expression in the gonad sheath cells (arrowheads). The red-framed insets are from different worms and show a similar vesicular pattern within the intestinal cells in N2 and *paqr-2* mutant worms.

FIG. S6

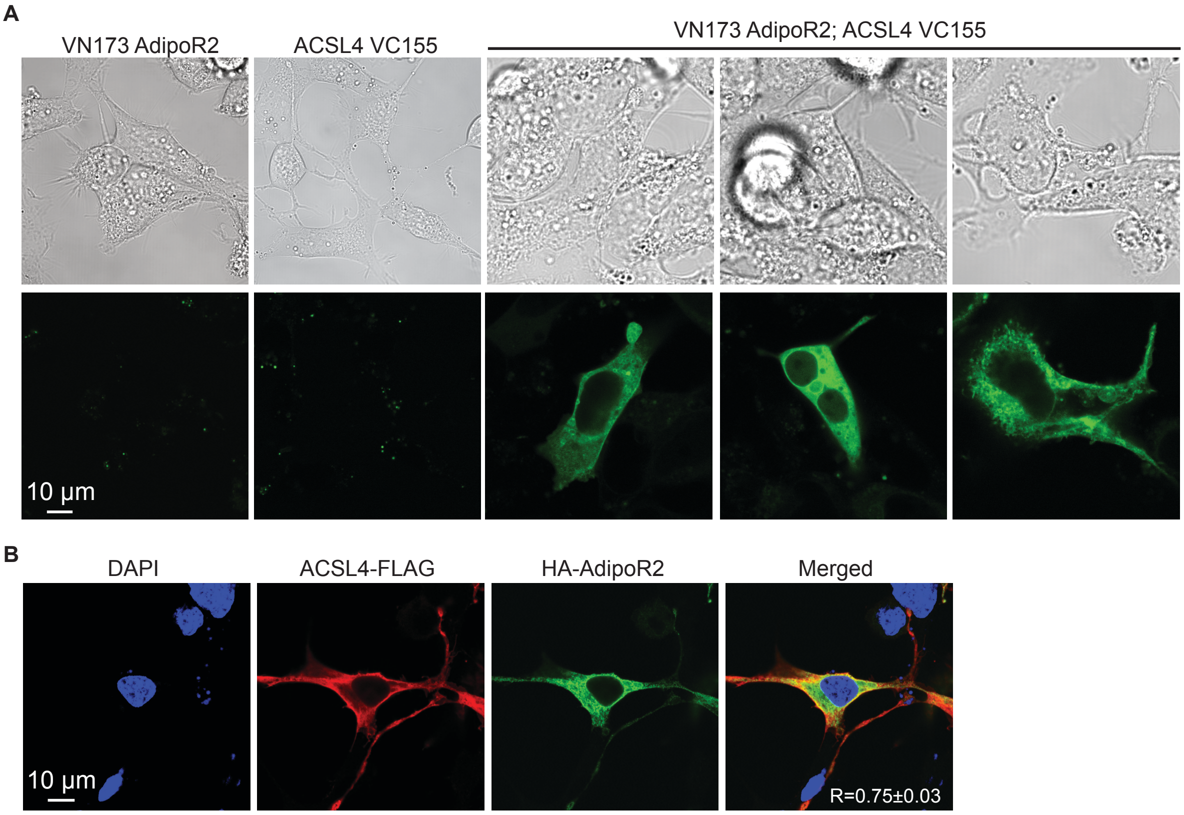

Suppl. Fig. S6. AdipoR2 interacts with ACSL4. (A) BiFC experiment images confirming that AdipoR2 interacts with ACSL4, with three additional examples of reporter co-expression. Note that the VN173 AdipoR2 panels are the same as in Fig. 4D. (B) Immunofluorescence confirming that HA-AdipoR2 co-localizes with ACSL4-FLAG. R is the Pearson correlation coefficient between the two fluorophores ± standard deviation (n=5).
